# Predicting Neuroblastoma Patient Risk Groups, Outcomes, and Treatment Response Using Machine Learning Methods: A Review

**DOI:** 10.3390/medsci12010005

**Published:** 2024-01-06

**Authors:** Leila Jahangiri

**Affiliations:** 1 School of Science and Technology, Nottingham Trent University, Clifton Site, Nottingham NG11 8NS, UK; leila.jahangiri@ntu.ac.uk; 2 Division of Cellular and Molecular Pathology, Addenbrookes Hospital, University of Cambridge, Cambridge CB2 0QQ, UK

**Keywords:** neuroblastoma, machine learning, multi-omics, classification, risk, outcome, survival, treatment

## Abstract

Neuroblastoma, a paediatric malignancy with high rates of cancer-related morbidity and mortality, is of significant interest to the field of paediatric cancers. High-risk NB tumours are usually metastatic and result in survival rates of less than 50%. Machine learning approaches have been applied to various neuroblastoma patient data to retrieve relevant clinical and biological information and develop predictive models. Given this background, this study will catalogue and summarise the literature that has used machine learning and statistical methods to analyse data such as multi-omics, histological sections, and medical images to make clinical predictions. Furthermore, the question will be turned on its head, and the use of machine learning to accurately stratify NB patients by risk groups and to predict outcomes, including survival and treatment response, will be summarised. Overall, this study aims to catalogue and summarise the important work conducted to date on the subject of expression-based predictor models and machine learning in neuroblastoma for risk stratification and patient outcomes including survival, and treatment response which may assist and direct future diagnostic and therapeutic efforts.

## 1. Introduction

Neuroblastoma (NB) is the second most common malignancy in infants and children, presenting as a tumour of the sympathetic nervous system. Circa 60% of these tumours occur in the abdominal region, and of those, half are located in the medulla of the adrenal glands [1,2].

NB staging is based on the international neuroblastoma risk group staging system (INRGSS) and relies on image-defined risk factors (IDRF), in which locoregional tumours (L1 and L2) display the absence and presence of IDRF, respectively. IDRFs represent surgical risk factors that can be identified in medical images. M displays disseminated disease, and MS encompasses L1 and L2 with metastasis limited to locations such as the skin, liver, and bone marrow (but not cortical bone) in infants younger than 1.5 years (18 months) [3,4].

Previously, the international neuroblastoma staging system (INSS) was utilised and included stages 1 and 2, which encompass locoregional tumours that can be completely or partially resected, respectively, while stage 3, which crosses the midline, is unresectable and unilateral and may or may not involve local lymph nodes. Stage 4 describes distant metastasis of any primary tumour, and 4S represents stages 1 or 2 tumours with limited metastasis to the liver, skin, and bone marrow (but not cortical bone) in children younger than the age of 12 months [3,4].

Risk stratification in NB patients divides cases into low, intermediate, and high-risk groups based on multiple parameters such as histological features, MYCN amplification, DNA ploidy, age, chromosomal alterations (including alterations to 11q), differentiation status, and stage at diagnosis [5,6,7,8,9]. Risk groups bear substantial differences in prognoses; for example, low- and intermediate-risk groups may display an overall survival (OS) of 95%, while high-risk cases will display a dismal survival of less than 50% and may experience a higher rate of recurrence despite treatment [5,6,7,8]. On a molecular level, high-risk MYCN amplification constitutes circa 40–50% of this category, while the alternating length of telomerase (ALT) and the arrangement of the telomerase reverse transcriptase (*TERT*) gene also constitute 25% and 23–31% of high-risk cases, respectively [10,11].

Big data technologies have shown great promise to process data from various sources in the form of structured and unstructured data, and have yielded clinically valuable information. These technologies have also led to the proposition and improvement of predictive models of patient prognosis and survival [12]. Many studies have used machine learning (ML) and statistical methods to develop multi-gene models or other predictor models to anticipate patient subgrouping, risk, outcome (including survival, relapse, and recurrence), and treatment response [13]. It is important to note that patient outcomes refer to the destination of the clinical intervention used, which will provide feedback on its efficacy, and therefore patient survival and mortality rate would be viewed as a subset of the patient outcomes [14].

One example of a multi-gene predictor model is a study that identified a 59-gene signature to predict NB patient survival [15]. This study built a gene-expression signature based on 30, 313, and 236 training, testing, and validation samples, respectively (579 patient samples in total). The resulting signature showed performance, sensitivity, and specificity of 85.4%, 84.4%, and 86.5%, respectively, in predicting the patient outcome [15]. Also, patients with a higher risk signature would be deemed at higher risk of death and relapse, with an odds ratio for overall survival (OS) and progression-free survival (PFS) of 19.32 and 3.96, respectively, suggesting that the 59-gene signature predict NB patient outcomes [15].

**Figure 1:**
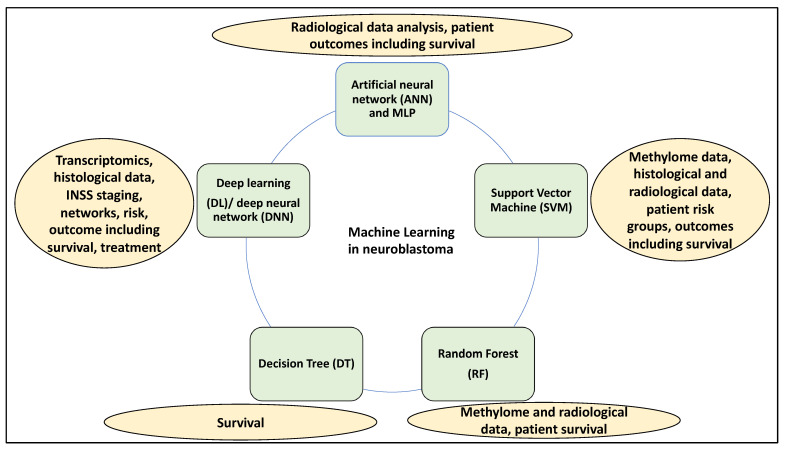
ML in NB. Various patient big data, including genomics, radiology, histology, and patient information, can be processed by ML methods such as artificial neural networks (ANN) and multilayer perceptron (MLP), which is a feedforward ANN architecture, support vector machines (SVM), random forest (RF), decision tree (DT), Deep learning (DL)/deep neural network (DNN). The tasks that can be used by ANN/MLP are the analysis of radiological/imaging data and can be used to predict outcomes, including survival. SVM can be used for methylome, histological, and radiological images and can be used to predict risk and outcomes, including survival. RF can analyse methylome data and radiological images and predict patient survival; DTs can be used to predict survival; and finally, DL/DNN can be used for transcriptomics and histological data. It can also be used for predicting INSS staging, risk groups, and patient outcomes including survival, treatment and network analysis. Notably, SVM can be used with SIFT or RFE, for histological data classification and methylome data, respectively.

Moreover, ML has been applied to a multitude of patient data, including multi-omics (genomic, transcriptomic, and methylome-sequencing data) in addition to microarray data [12,16] and histological and medical imaging data [17,18] to establish risk and predict outcomes and survival and treatment outcomes. ML methods include support vector machines (SVM) [19], artificial neural networks (ANN) [20], decision tree (DT) [21], and random forest (RF) [22]. Consistently, SVMs are used for classification and regression in a supervised fashion [23]. ANN is an adaptive self-learner that imitates biological networks’ behavioural properties [24], while multilayer perception (MLP) is a feedforward ANN architecture that is known to be a robust approximator for classification and prediction purposes and may be viewed as the most commonly used ANN system [25]. Further, DTs, on the other hand, recursively separate observations made into branches to generate a tree to improve prediction and decision accuracy [21,25]. RF is an ensemble ML algorithm that can process complex interactions and classification features, learns rapidly, and performs robustly even with missing data [26]. Moreover, logistic regressions are used to make binary or multi-class variable predictions and are borrowed by ML methods to predict the probabilities of classes [27]. Deep learning (DL), as a subsection of ML, is generally used synonymously with deep neural networks (DNN) and can transform data through various representation levels. DL (DNN) can also predict clinical outcomes in NB patients [28], and it can be utilised for image detection and analysis in cancer [29]. Also, convolutional neural networks (CNN), as a type of DL (DNN), have been shown to learn patterns within images for classification and object recognition [27] (Figure 1). Finally, ML can be supervised or unsupervised; in the supervised fashion, the input data is labelled, while it is not in the unsupervised learning method.

Many studies use ML tools for patient outcome prediction. For example, Oberthuer et al. have shown that SVM can be utilised to classify high-risk NB patients based on 4 × 44 K microarray data from 709 NB samples. The classification model was built on 75 NB tumours with contrasting characteristics and clinical courses, while validation was performed on the remaining 634 samples, and Kaplan–Meier and multivariate Cox regression were also utilised [23]. The classifier generated predicted patient outcomes with an accuracy of 0.95, and this showed the highest clinical value for low and intermediate-risk patients (low-risk, event-free survival (EFS): 0.84, OS: 0.99, and intermediate-risk, EFS: 0.88 and OS: 1 for these groups, respectively). This method could be integrated into the risk stratification system for low- and intermediate-risk patients [23].

Given this background, in this study, the utility of multi-omics, histological, and radiological data for ML processing was discussed. Finally, studies reporting data processing by ML to stratify risk groups and predict outcomes, survival, and treatment response were examined.

## 2. The Use of Patient Data for Predicting Patient Outcomes

### 2.1. The Use of Expression-Based Data for the Development of Multi-Gene Predictor Models and INSS Staging

Expression-based data, including RNA-sequencing, and microarrays, can be useful for the molecular classification of cancers and for identifying biomarkers, drug targets, and patient prognosis [16]. Three putative genomic subtypes have been proposed in NB, inclusive of types 1, 2A, and 2B. Accordingly, type 1 displays high TrkA expression, low-risk tumour, and triploid DNA content; 2A comprises intermediate-risk tumours, frequent 11q deletions, and 17q gain with no MYCN-amplification; while 2B comprises high-risk tumours with MYCN amplification, a high frequency of 1p deletions, and 17q gains [30,31]. The most aggressive NB type comprises MYCN amplification and low expression of NTRK1, while elevated expression or mutations in ALK are also linked to unfavourable outcomes [32]. This study explored subgroups based on the gene expression profiles of 47 microarray samples, and 4 subgroups (p1–p4) were identified within these 2 separate datasets using principal component analysis and verified using unsupervised hierarchical clustering of a third dataset (comprising 101 NB samples) [32]. For example, the p3 subgroup was significantly linked to MYCN amplification and 1p deletion, while INSS stages 3 and 4, unfavourable outcomes, and 17q gain were linked to subgroups p2, p3, and p4, respectively, rather than p1. The occurrence of the 11q deletion was higher in the p2 and p4 groups [32].

**Figure 2:**
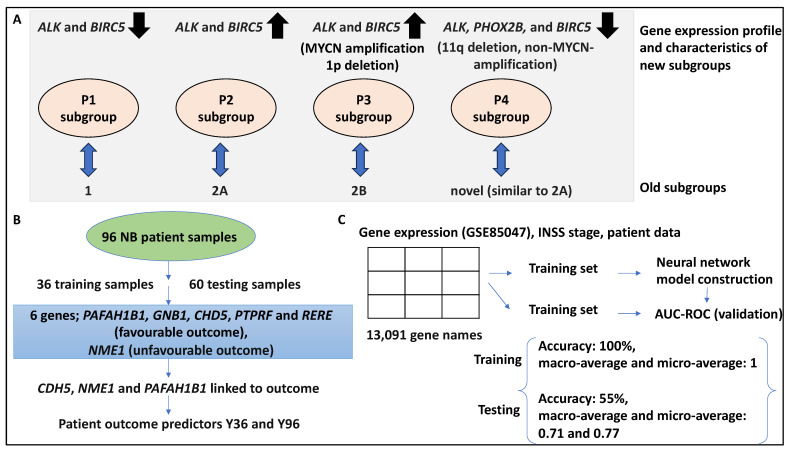
The use of expression-based data for the development of multi-gene predictor models and INSS staging. (**A**) The established subgroups of NB comprise 1, 2A, and 2B, in which the 1 subgroup includes Trk expression, ploidy, and low-risk stratification; 2A includes intermediate-risk stratification, 11q deletion, and 17q gains but no MYCN-amplification; and finally, 2B includes MYCN-amplified samples with a high frequency of 1p deletion and 17q gains. The study classified NB samples based on 47 microarray samples into 4 subgroups (p1–p4); this was verified using 101 NB samples. For example, p3 was linked to MYCN amplification and 1p deletion, while p4 was linked to 11q deletion and non-MYCN amplification. As for gene expression, *ALK* and *BIRC5* were upregulated in subgroups p3 and p2, while these genes were downregulated in p1 and p4. The p4 subgroup was novel and showed some features of 2A. P4 also showed an ALK expression reduction. (**B**) of the 96 NB samples, 36 were utilised to develop a gene expression-based model, and 60 were used to test the model. 11 normalised gene expression levels were z-transformed and analysed using Cox regression, and of these only 6 genes, 5 (*PAFAH1B1*, *GNB1*, *CHD5*, *PTPRF*, and *RERE*) were found to be statistically significantly linked with favourable patient outcomes, while *NME1* was linked to unfavourable clinical outcomes. Using principal component analysis and Cox regression in a backward selection fashion, *CDH5*, *NME1*, and *PAFAH1B1* were found to be strongly linked to outcomes and this formed the basis of the Y36 outcome prediction score. The 60 testing samples were also successfully distinguished by the outcome predictor, and the predictor model was updated to Y96. 352 patient gene expression datasets were tested using the Y96 predictor model, yielding two groups with distinct OS and EFS values. (**C**) A study used the gene expression data of the GSE85047 dataset, INSS staging information and patient data to form matrices that were then fed to the DNN architecture. This matrix contained 280 patients and 13,091 gene names, and these were split by training and testing datasets. The accuracy, macro-average, and micro-average values for each dataset were then calculated. The accuracy of the training and test datasets was 100% and 55.56%, respectively. Also, the macro-average and micro-average AUCs were calculated as 1 for the training dataset, and 0.71 and 0.77 for the testing dataset, respectively.

The authors then performed literature mining and found that the expression of six genes, including *ALK*, *BIRC5*, *MYCN*, *CCND1*, *NTRK1*, and *PHOX2B*, distinguished four clusters. For example, *ALK* and *BIRC5* were upregulated in subgroups p3 and p2, while these genes were downregulated in p1 and p4. MYCN was upregulated five times in p3 compared to p1 and p4. These subgroups also displayed differential Kaplan–Meier-based OS and EFS; for example, p3 and p4 showed an OS of 50% and 62.5%, while the EFS for these two subgroups was 22.2% and 25%, respectively [32]. This subgrouping could be tested by unsupervised hierarchical clustering in a testing and validation dataset comprising 101 NB samples, and accordingly, 90 samples clustered into 4 hierarchical clusters of h1–h4 similar to p1–p4 [32]. Overall, the clusters identified by principal component analysis yielded p1, p2, and p3 groups, which corresponded to the genomic subgroups (1, 2A, and 2B, respectively), while a new cluster was also found in all datasets (p4) that, in some cases, was similar to 2A but in other cases was similar to the other subgroups [32]. This fourth subgroup comprised samples with 11q deletion, non-MYCN-amplification status, and reduced expression of *ALK*, *PHOX2B*, and *BIRC5*, and interestingly, this group was linked to unfavourable patient outcomes and reduced survival [32]. In conclusion, this study identified a 6-gene signature for NB classification (Figure 2A).

In another study, the authors attempted to develop quantitative RT-PCR-based predictors of NB patient outcomes using 96 NB samples [33]. This outcome predictor model utilised 11 differentially expressed genes. Accordingly, of the 96 samples, 36 were utilised to develop a gene expression-based model, 60 were used to test the model, and an additional 120 NB samples were used to validate the model by RT-PCR. Of the 36 training datasets used for qRT-PCR studies, 11 normalised gene expression levels were initially z score-transformed and analysed using Cox regression, and of these, only 5 (*PAFAH1B1*, *GNB1*, *CHD5*, *PTPRF*, and *RERE*) were found to be statistically significantly linked with favourable patient outcomes, including longer EFS and OS, while *NME1* was linked to unfavourable clinical outcomes [33]. Furthermore, having applied principal component analysis and univariate Cox regression in a backward selection fashion, only *CDH5*, *NME1*, and *PAFAH1B1* were found to be most robustly linked to OS and EFS, and this formed the basis of the Y36 outcome prediction score. Accordingly, low and high Y36 levels were linked to decreased and increased patient survival, respectively. The 60 testing NB samples were also utilised to study these 3 genes (i.e., *CDH5*, *NME1*, and *PAFAH1B1*) and the outcome predictor could distinguish 2 groups with significant differences in OS and EFS (HR, 9.3 and 3.1, respectively) [33]. The model was then updated to capture all the 96 NB samples used and was therefore referred to as the Y96 predictor model.

In addition, 352 patient gene expression datasets obtained from microarrays (namely set 2, n = 101, and set 3, n = 251) were used for further validation using the Y96 predictor model [33]. The predictor model accurately separated these patients into two groups with distinct OS and EFS levels. For example, set 2 had a 5-year OS of 0.97 (HR = 10.5) and a 5-year EFS of 0.91 (HR = 13.3), while set 3 had a 5-year OS of 0.99 (HR = 28.1) and a 5-year EFS of 0.96 (HR = 15.6). Further, for set 2, only one patient with low-risk classification (stage 3 with no MYCN amplification) was misclassified and suffered a fatal disease, while for set 3, 7/148 patients had an event despite being assigned to the low-risk group [33]. Finally, the AUC for the ROC test was shown to be 0.87 and 0.89 for OS and EFS for the outcome predictors proposed in this study. In conclusion, the authors showed that *CDH5*, *NME1*, and *PAFAH1B1* genes formed a 3-gene signature predictive of patient outcomes (Figure 2B) [33]. 

A study attempted to link gene expression profiles with INSS stage prediction by using a DNN [28]. Accordingly, the DNN was constructed based on gene expression omnibus (GEO) and the cancer genome atlas databases (TCGA), which, in association with patient information and INSS staging, could allow the examination of correlations between genomic data and clinical parameters [28]. Consistently, 280 NB datasets deposited in GSE85047 and its associated clinical data were obtained, and matrices inclusive of INSS stage and gene expression array data were formed and fed to the DNN architecture [28]. This matrix contained 280 patients and 13,091 genes and was divided by training and testing datasets (by a ratio of 8:2), and the accuracy of the model was calculated for both datasets and reported as AUC-ROC curves. For example, after 5000 iterations, the accuracy of the approach for training and test datasets was 100% and 55.56%, respectively. Also, the macro-average, micro-average, and all the AUC values for the estimation of one-versus-rest (OVR) were calculated as 1 for the training dataset. Also, for the testing dataset, the macro-average and micro-average AUCs were 0.71 and 0.77, respectively [28]. The OVR AUCs for patient stages ranging from 1–4S were 0.8, 0.66, 0.59, 0.85, and 0.58, respectively. Overall, the poor AUC for the test datasets may have been due to overfitting for stages 2, 3, and 4S, and this model distinguished stage 1 and 4 patients (alternatively, there may not have been distinguishable genes between 2, 3, and 4S stage expression profiles) [28]. In conclusion, the DNN model developed by this study could be improved by adding more labelled samples (Figure 2C).

In summary, this section suggested that transcriptomics efforts (including microarray and RNA-sequencing) can be analysed for the establishment of multi-gene predictor models capable of discriminating amongst NB subtypes. To this end, unsupervised expression profiling can be achieved by principal component analysis followed by unsupervised hierarchical clustering, and this approach can distinguish between subtypes. In addition, differentially expressed genes tested by qPCR could be z-transformed and analysed by univariate Cox regression and principal component analysis in a backward selection fashion, yielding a subset of genes linked to various clinicopathological aspects such as OS and EFS. Using these methods, it is possible to form a useful multi-gene one-score predictor model of outcome (for example, Y96). DNN can be applied to NB expression data from the GEO and TCGA databases to accurately classify patients based on the INSS stage.

### 2.2. Methylome Data to Predict MYCN Status-Linked Outcomes

NB is, for the most part, epigenetically regulated [34,35], and the CpG island methylator phenotype (CIMP) is linked to poor NB patients; therefore, it stands to reason that methylation signatures may indeed be of significant interest for NB prognosis studies [36]. In a study, the link between CpG methylation signature and MYCN amplification status was investigated in a total of 126 NB samples, inclusive of 45 MYCN-amplified and 81 non-MYCN-amplified datasets deposited to therapeutically applicable research to generate effective treatment (TARGET); from these datasets, 396,065 CpGs were retrieved [37]. Differential methylated CpGs were obtained using the ChAMP programme, and the authors found 663 differentially methylated CpGs of 369 differentially methylated genes. Of these 369 differentially methylated genes, 238 and 131 genes displayed high and low methylation status, respectively, in MYCN-amplified compared to non-MYCN-amplified samples [37]. Additionally, 14 genes, including *NXPH1* and *SOX2-OT*, were methylated at high levels (defined as having at least six differentially methylated CpGs) between MYCN-amplified and non-amplified samples. Furthermore, gene ontology analyses showed the enrichment of the extracellular matrix and neural crest differentiation term, suggesting links to NB [37].

Further, recursive feature elimination (RFE) was utilised to retrieve the most important features. Accordingly, 25/663 of these islands were processed by RFE and ML (SVM), and these CpG enabled a 100% clustering accuracy of these 126 TARGET samples based on their MYCN amplification status (with no misclassification) [37]. Similarly, a dendrogram plot was generated using ensemble hierarchical and K-means clustering applied to the 663 CpGs. This led to the correct clustering of the 122/126 TARGET datasets [37].

The 25/663 CpG feature selected by RFE was utilised to build an SVM ML model to classify the 126 TARGET samples. This model was then tested on three testing datasets (GSE54719 (n = 35), GSE65306 (n = 28) and GSE120650 (n = 58)) obtained from the GEO, yielding accuracies of 100, 93, and 97%, respectively [37].

The study also utilised Kaplan–Meier survival plots and Cox regression. Furthermore, specific CpGs associated with patient survival were also established [37]. Accordingly, eight CpGs showed links to survival, while five CpGs were associated with EFS. For example, cg00540828, linked to the *CUX1* gene, showed a coefficient of −0.0146 and was linked to OS. Also, cg01710189 loci, linked to the *PDLIM2* gene, displayed a coefficient of −1.254 and was associated with EFS. It is noteworthy that positive coefficient CpGs were linked to the MYCN-amplified category, while negative coefficient CpGs were linked to the MYCN non-amplified category [37].

**Figure 3:**
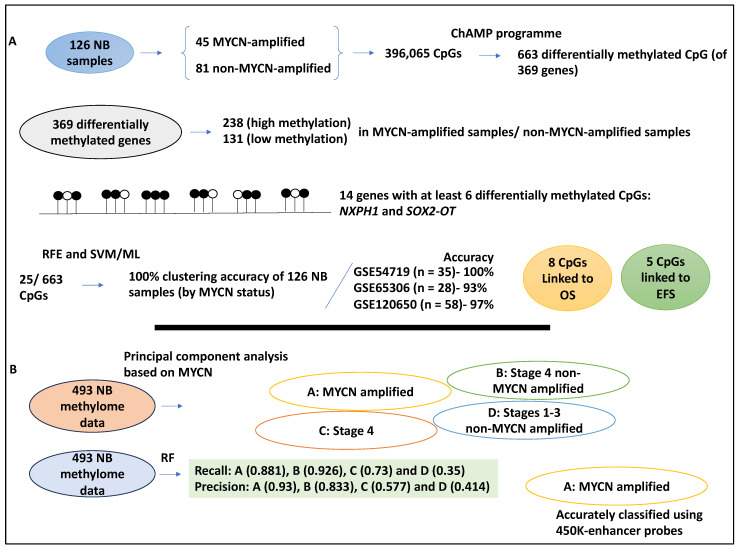
Applying ML methods to methylome data to predict MYCN status-linked outcomes. (**A**) 128 NB samples with methylation information were split into 45 MYCN-amplified and 81 non-MYCN-amplified datasets, and 369,065 CpGs were obtained. Using the ChAMP programme, 663 differentially methylated CpGs pertinent to 369 genes were obtained. Of these 369 differentially methylated genes, 238 and 131 had high and low levels of methylation in MYCN-amplified samples compared to non-MYCN-amplified samples. 14 genes, including *NXPH1* and *SOX2-OT*, showed at least 6 differentially methylated CpGs. 25/663 features were deemed most important, and after processing these features using RFE and ML, 100% accuracy of MYCN amplification status (amongst the 126 datasets) was achieved. This model was then tested on three testing datasets (GSE54719 (n = 35), GSE65306 (n = 28) and GSE120650 (n = 58)) yielding accuracies of 100, 93, and 97%, respectively. Finally, 8 CpGs showed links to OS, while 5 CpGs were associated with EFS. (**B**) The 493 NB methylome data (Human Methylation 450 K dataset), was processed using principal component analysis, and it clustered with MYCN status and yielded 4 groups. The 4 clusters of patients included: A, MYCN-amplified patients; B, stage 4 INSS without MYCN amplification; C, stage INSS 4 patients; and D, stages 1–3 without MYCN amplification. RF was applied to this dataset and for group A-D, the recall was 0.881, 0.926, 0.73, and 0.35, respectively, while precision was 0.93, 0.833, 0.577, and 0.414, respectively. MYCN-amplified group A was accurately classified when 450 K-enhancer probes were used.

Kaplan–Meier analyses also showed that MYCN amplification status was associated with CpG score and patient survival (OS, hazard ratio, HR = 5.11, EFS, HR = 4.84) since CpG high and low scores were linked to MYCN amplification and non-amplification, respectively [37]. Notably, CpG scores were based on their coefficients and were given a value of 1 or 0 for each patient. In conclusion, this study utilised statistical and ML methods to extract features of DNA methylome data to retrieve signatures predictive of MYCN amplification status and patient prognosis, which may be useful for early patient diagnosis and risk stratification (Figure 3A) [37].

Another study attempted to understand DNA methylation as part of the epigenetic mechanisms of gene regulation in NB by utilising RF [38]. RF is simple, intuitive, and computationally less costly, and this study attempted to identify an intermediate-risk group within the low-risk subpopulation of NB patients [38]. RF could be slightly slow in forming a model once trained. Accordingly, 493 NB methylome data, referred to as the Human Methylation 450 K dataset, was collected from TARGET and other sources. Initially, the data was processed using principal component analysis, and it was shown that MYCN amplification formed a cluster, unlike the INSS stage, which did not show a clear cluster. This suggested that perhaps MYCN amplification status was linked to the DNA methylation pattern [38]. Moreover, this analysis distinguished 4 clusters of patients: A, MYCN-amplified patients; B, stage 4 INSS without MYCN amplification; C, stage INSS 4 patients; and D, stage 1–3 without MYCN amplification [38].

The authors also evaluated the adequacy of the classification method of group A–D by applying RF to the Human Methylation 450 K dataset. For groups A and B with more high-risk characteristics, the recall was much better (0.881 and 0.926) than the low-risk groups C and D (0.73 and 0.35), respectively, indicating that groups A and B were linked to alterations in DNA methylation. In addition, the authors established precision scores for samples, and it was shown that groups A and B (0.93 and 0.833, respectively) were correctly and accurately classified; this did not stand true for groups C and D (0.577 and 0.414, respectively) since circa half the samples were accurately predicted [38].

Moreover, the feature selection aspect utilised in this study was based on probe annotation since the number of samples (n = 493) was fewer than the variables (*p* > 480,000). Expert knowledge about the transcription start site, enhancer, and CpG islands was used to design probes. The prediction power of these probe annotation groups was expressed in metrics like the F1-score. For example, the F1-score for 24 probe groups, including but not limited to the promoter, transcription start site, 3′-untranslated regions, and CpG islands within the regions, was plotted for groups A and B. Group A was accurately classified when 450 K-enhancer probes were used, but promoter regions bearing CpG islands showed lower prediction capacity [38]. This suggested that enhancer-annotated regions had strong predictive power, and this was more profound for MYCN-amplified NB.

In conclusion, this study showed that the MYCN-amplified A group was linked to DNA methylation of the enhancer regions. In addition, this RF-based model showed high predictive power compared to other models (Figure 3B) [38].

In summary, 25/663 CpGs can be selected by RFE and used to build the SVM model capable of stratifying NB patients based on MYCN amplification status, wherein these unique CpGs may be useful for building a diagnostic indicator for MYCN amplification in this cancer. Cox regression can be used to assess the impact of these 25 CpGs on NB patient survival. Finally, analysing NB methylome data from TARGET with principal component analysis can yield clusters of patients based on MYCN amplification status and the adequacy and accuracy of the approach for each of the four clusters (subgroups) can be established using RF. Subgroup classification based on probe annotation may also be relevant, and enhancer probes may display a higher level of accuracy compared to other loci. Overall, this section outlined the study of methylome data using ML for predicting subgroups based on MYCN status.

### 2.3. Histological Data for ML to Assist NB Diagnostics

**Figure 4:**
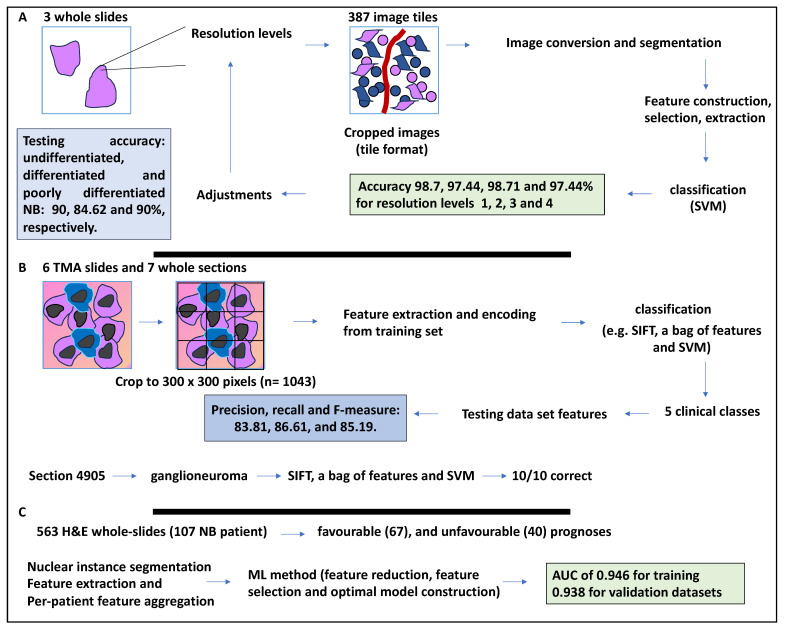
The use of patient histological data for ML-based classification. (**A**) A study used 3 whole H&E slides of neuroblastic cancer to retrieve 387 image tiles so that they were a good representation of the discriminating features. The authors used a leave-one-out method to select features for each resolution level. For example, feature extraction followed by SVM-based classification yielded 3, 6, 10, and 5 features for resolution levels 1, 2, 3, and 4, respectively. For the testing dataset, inclusive of 33 whole slides, accuracies of 90, 84.62, and 90% were obtained for undifferentiated, differentiated, and poorly differentiated NB classes, respectively. (**B**) This study used 6 TMA slides and 7 whole sections from 125 NB patients. H&E-stained tissue slides (under 5 categories of undifferentiated NB, poorly differentiated NB, differentiating NB, ganglioneuroma, and ganglioneuroblastoma). These images were cropped into 300 × 300 pixels, representing key features of subtypes (a total of 1043 cropped images, 5 subtypes, and 125 patients). Having extracted features, the SVM classifier could classify five clinical classes. Testing datasets were also processed. Overall, by using SIFT, a bag of features, and SVM, precision, recall, and F-measure were 83.81, 86.61, and 85.19, respectively. For testing, they used 5 whole slides and applied the described method, yielding a tissue section (4905) of ganglioneuroma, and 10/10 sub-images were assigned as this. (**C**) 563 H&E whole-slides were obtained from 107 NB patients, including 2 groups of favourable (67) and unfavourable (40) prognoses. Processing included nuclear instance segmentation, feature extraction, and per-patient feature aggregation. The ML method for prognosis prediction included feature reduction, feature selection, and model construction. The AUC for the training and validation datasets were 0.946 and 0.938, respectively.

The management of NB can be greatly improved by histopathological classification, and histopathological studies are considered the gold standard for NB diagnostics and classification [39]. Pathologists use microscopes to classify tumour samples deposited onto glass slides and stained, usually with H&E stain, and these slides are then examined at low and then high magnification in search of representative regions that may assist in the NB classification [39]. As such, the NB grading process requires a trained pathologist to accurately determine the morphological properties of the tumour tissue using a microscope [17]. However, digital scanners have enabled the scanning of tissue samples in a cross-sectional fashion to obtain whole-slide digital scans. As a result, these images can allow for qualitative and quantitative parameter evaluation [17]. A study by Kong et al. in 2009 applied image analysis to H&E-stained slides of neuroblastic cancer patients. Texture features obtained from tissue-segmented components were extracted and processed by a classifier previously trained by training images comprising various neuroblastic differentiation grades. Accordingly, the training dataset consisted of 387 image tiles obtained from three whole slides. This approach yielded a good representation of the input discriminating data, and on a plot, each class was compact and defined. The selected features were then analysed by different classifiers for each resolution level over the training datasets using a leave-one-out validation strategy. In this method, all samples except one were used for training, and the left-out sample could then be used for testing. For example, feature extraction followed by classification by SVM for resolution levels 1, 2, 3, and 4 yielded 3, 6, 10, and 5 features, respectively [17]. Further, the authors obtained the accuracy of this classification on a tile level. For example, SVM for resolution levels 1, 2, 3, and 4 yielded 98.7, 97.44, 98.71, and 97.44% accuracy, respectively. The authors also established classification accuracy per 33 whole slides as testing datasets. These neuroblastic types, comprising 10 undifferentiated NB, 13 differentiated NB, and 10 poorly differentiated NB, yielded accuracies of 90, 84.62, and 90%, respectively, and the overall accuracy was 87.88%; therefore, the tool was promising for improving NB grading (Figure 4A) [17].

Computer-aided diagnostics (CAD) is gaining momentum to assist in patient classification, which was adapted to the current study [40]. The classification methods from NB histological images relied on segmentation and feature extraction. Segmentation relies on morphological features, including the shape and size of cells, while extracting key features of NB images may allow the recognition of features that are not easily noticeable by the eye [39]. The study aimed to combine Scale Invariant Feature Transform (SIFT) (robust to scale variation) with an encoding algorithm to retrieve distinct features from the images. The use of the bag of features reduced the number of features extracted [39]. Accordingly, the authors used images of NB, including six H&E-stained tissue microarrays (TMA) slides retrieved from the tumour bank of a children’s hospital in Australia and seven whole sections for 125 NB patients. The diameter of the TMAs was in the region of 1.2 mm, and a thickness of 3 µm stained with H&E. These samples were initially classified into (1) undifferentiated NB, (2) poorly—differentiated NB, (3) differentiating NB, (4) ganglioneuroma, and (5) ganglioneuroblastoma [39]. To reduce the size of the images, the images were cropped into regions of 300 × 300 pixels, in which the cropped regions contained all the specifications of a subtype; this amounted to 1043 cropped images under the five described subtypes for a total of 125 patients. The 1043 cropped images were split into three datasets: 623 for training, 209 for testing, and 211 for the second section of the validation process. Having extracted distinct features and fed them to the encoding block to refine them into discriminative features, SVM then classified the images into five clinical classes. Overall, the algorithm (SIFT-descriptor) extracted feature vectors consisting of 128 aspects [39].

The authors reported contrast and edge thresholds for the SIFT process and relevant classification accuracies. Contrast and edge thresholds eliminated key points bearing low contrast and unstable key points near edges, respectively [39]. For example, for a contrast threshold of 0.04, the classification accuracy was 76.58%, and for an edge threshold of 11, the classification accuracy was 81.76%. The authors then tested the remaining training set and the testing set, repeated the analyses 10 times, and reported the average accuracy. The proposed method comprising SIFT with the bag of features and the SVM classifier outperformed other methods since the precision, recall, and F-measure were 83.81, 86.61, and 85.19%, respectively [39].

Finally, 623 sub-images from the Australian dataset were used for training, and five whole tissue sections from Bristol, consisting of one ganglioneuroblastoma, one ganglioneuroma, three poorly differentiated NB, and ten randomly selected sub-images from each whole image were used for validation. The algorithm first assigned a label and then classified them using a majority vote of 10 sub-images [39]. For example, a tissue section (4905) was ganglioneuroma, and 10/10 sub-images were assigned as this. In conclusion, the proposed method was viewed as a useful method for H&E-based predictions that may facilitate diagnostics (Figure 4B) [39].

Moreover, the diagnosis and prognosis of NB are largely assisted by the international neuroblastoma pathology classification (INPC). Although there may be inconsistencies in the analysis of patient samples by pathologists, a study sought to establish a method of reducing variability in classification. To that end, 563 H&E whole-slides were obtained from 107 NB patients who had undergone surgery for tumour resection, and two distinct groups of favourable (67) and unfavourable (40) prognoses were established [41]. Accordingly, the authors streamlined the multiple processes of nuclear instance segmentation, feature extraction (including nucleus-level morphological and intensity feature extraction), and per-patient feature aggregation [41]. For example, after nuclear instance segmentation, the number of nuclei, those nuclei identified by the algorithm, and false positives were established as 3408, 3407, and 46, respectively. That would represent a recall and precision of 98.62% and 98.65%, respectively. The next step was the ML method for prognosis prediction, which included feature reduction, feature selection, and optimal model construction. For example, patient-level features were reduced to 25, after which the datasets were split by training and testing and underwent bootstrap resampling (1000×) [41]. A logistic regression was also used to incorporate features into a multivariate model, and then various feature combinations were tested and AUCs were generated. Optimal model construction also entailed boot-strapping resampling (1000×) with adjustments. For example, in both the training and testing datasets, parameters such as age and nucleus morphology intensity of features could accurately classify the patients (AUC of 0.946), while the validation dataset achieved an AUC of 0.938 (Figure 4C) [41].

In conclusion, this study suggested that features derived from images, including nuclear morphology, could be prognostic, and therefore this feature could assist pathologists with more accurate classifications.

Overall, this subsection highlighted the use of ML and statistical methods to facilitate NB diagnostics predicated on histology slides. Feature extraction from tissue-segmented components of scanned slides can be analysed using a classifier trained on images of neuroblastic differentiation grades. For example, a training dataset might include image tiles obtained from whole slides, which can then be analysed by a classifier (such as an SVM) for multiple resolution levels using a leave-one-out approach and the accuracy of the approach can be calculated. Also, the use of SIFT can assist in the retrieval of distinct features that are robust to scale variation from images, while using the bag of features can reduce the number of features. The method can obtain good precision, recall, and F-measure. Finally, nuclear instance segmentation, feature extraction, and per-patient feature aggregation can be conducted, and this can be followed by feature reduction, selection, and model construction (ML), and good accuracy can be obtained.

### 2.4. Radiological Data for Clinical Predictions

Radiological patient data can be processed by ML [18]. As such, a study analysed radiomics based on CT scans to establish intra-tumoural heterogeneity, relying on specific statistical features within an image obtained from the entire primary tumour volume [18]. Accordingly, the authors hypothesised that CT-based radiomics features were linked to heterogeneity, and, through that, to patient outcomes including IDRF, tumour differentiation, metastasis, and MYCN amplification status. To that end, the authors used six ML tools (radiomics-based ANN, Lasso and elastic-net regression, RF, and SVM) to retrospectively process medical images and link them to patient outcomes. For example, an ANN method was used to extract tumour radiomics features from the CT scans, and the other ML tools were also trained alongside this and then tested for various patient outcomes. This study also used a pre-trained 2D CNN on some images for comparison [18]. Nested cross-validation strategies were also implemented for splitting the datasets.

Of these 65 patients, 35 and 30 were obtained from two children’s hospitals, respectively. Primary tumours were segmented from CT scans and reviewed by a trained radiologist. A pyradiomics library was utilised to retrieve 105 radiomic features categorised into classes (including but not limited to 23 grey-level co-occurrence matrices and 16 grey-level run-length matrices) to characterise tumours [18]. The authors prevented overfitting by applying nested cross-validation approaches to split the training and testing datasets (for example, fivefold outer cross-validation and threefold internal cross-validation). Also, for the CNN, working with 2D slices of 3D images was a useful strategy to increase the training and testing dataset size 25-fold. In addition, each experiment (cross-validation process) was repeated 10 times to evaluate the ROC-AUC. For the CNN model, the mean prediction score of 25 images was calculated per patient, and then AUC for fivefold testing datasets were generated. As mentioned, six ML algorithms were utilised. For example, the authors formed a 3-layer ANN architecture with 1 and 10 hidden layers and units, respectively, to predict outputs including metastasis, differentiation grade, MYCN status, IDRFs, mortality, and mitosis to karyorrhexis index (MKI) [18].

**Figure 5:**
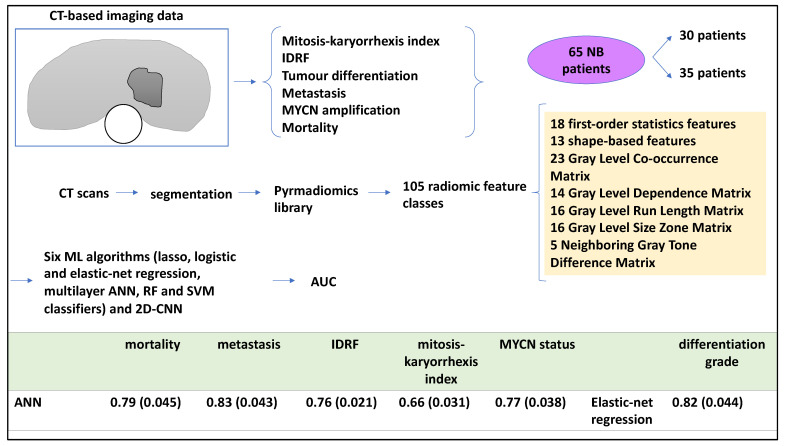
CT-based radiomics data used by ML for key clinicopathological factor predictions. CT scan-based image data was used in this study to establish factors such as MKI, IDRF, tumour differentiation, metastasis, MYCN amplification, and mortality. 65 patients, comprising 35 and 30 from 2 children’s hospitals. Primary tumour sections were segmented and reviewed; a pyramidomics library was also used to obtain 105 radiomic features, and these features were categorised into at least 7 categories. Six ML algorithms and a 2D-CNN model were utilised; overall, the authors formed a 3-layer ANN architecture. The table summarises the ROC-AUC values (with standard deviations) for the six clinicopathological aspects obtained by the best-performing algorithms.

ROC-AUC was also conducted, and the radiomics-based ANN, the best-performing model, obtained a ROC-AUC value (and standard deviation) of 0.79 (0.045) for mortality, while this algorithm also obtained 0.83 (0.043) for metastasis. For neuroblastic differentiation grade, elastic-net regression performed better and obtained a ROC-AUC value of 0.82 (0.044) [18]. As for secondary outcomes, the radiomics-based ANN obtained 0.76 (0.021), 0.66 (0.031), and 0.77 (0.038) for IDRF, MKI, and MYCN status, respectively. A pre-trained 2D CNN model performed worse when compared to the best-performing model proposed in this study; for example, for metastasis, a ROC-AUC of 0.77 (0.068) was obtained (Figure 5) [18]. 

In summary, the study revealed that ANN methods performed better than other algorithms (2D-CNN, Lasso and elastic-net regression, RF, and SVM) for predicting all the indicated aspects, except for the grade of differentiation from CT scans. In addition, it was possible to use nested cross-validation methods to split training and testing datasets repeatedly to prevent overfitting.

## 3. ML for 3 Critical Clinical Aspects (Risk, Outcomes including Survival, and Treatment)

The previous section dissected the use of various patient data for predicting patient outcomes. In this section, the topic was turned on its head and dissected the use of ML methods for the determination of patient risk, outcomes (including survival), and treatment.

### 3.1. ML to Determine Risk Stratification

In a study, the high-risk NB group was studied in greater detail using a DL (DNN) algorithm [42]. Accordingly, 407 high-risk NB samples were collected from TARGET, comprising 217 and 380 gene expression and copy number alteration datasets, respectively. Within this dataset, 190 samples had both expression and copy number alteration data and were used for training and identifying prognostic aspects. An extra 176 NB samples (SEQC) were used for external validation [42].

The authors stacked high-risk subtypes based on matrices of copy number alteration and gene expression of the 190 training samples. This information was transformed into 100 new features obtained from the autoencoder. The autoencoder (a DL method) comprised five layers of NN and three hidden layers (with 500, 100, and 500 nodes). Cox proportional hazard regression reduced these 100 new features to 35 that were linked to EFS and OS scores (*p* < 0.05). Using K-means clustering analysis, the 35 new features clustered with clustering numbers ranging from 2–6. The authors also used the C index to determine the optimal number of clusters, which is two (two clusters G1 and G2 were formed) [42].

Given this setup, the authors assessed the prognostic differences between high-risk subgroups, G1 and G2, in which G1 was assigned ultra-high risk. For example, the autoencoder could distinguish G1 and G2 based on EFS and OS readouts with *p* values of 2.2 × 10^−7^ and 2.8 × 10^−8^, respectively. The C-index for the autoencoder was 0.74 for EFS and 0.71 for OS. Notably, the principal component analysis and iCluster could also distinguish G1 and G2, and the autoinducer outperformed all other methods [42]. For example, principal component analysis obtained EFS and OS *p* values of 0.068 and 0.012, while iCluster, obtained EFS and OS *p* values of 1.22 × 10^−4^ and 3.76 × 10^−5^), respectively. As mentioned, the validation dataset was also considered, and four classifiers, including SVM, naïve Bayes, logistic regression, and XGBoost, were utilised for the classification and prognostic prediction. SVM performed better than the other three classifiers (average AUC of 0.844) and was also able to split the high-risk cases into two subgroups [42].

**Figure 6:**
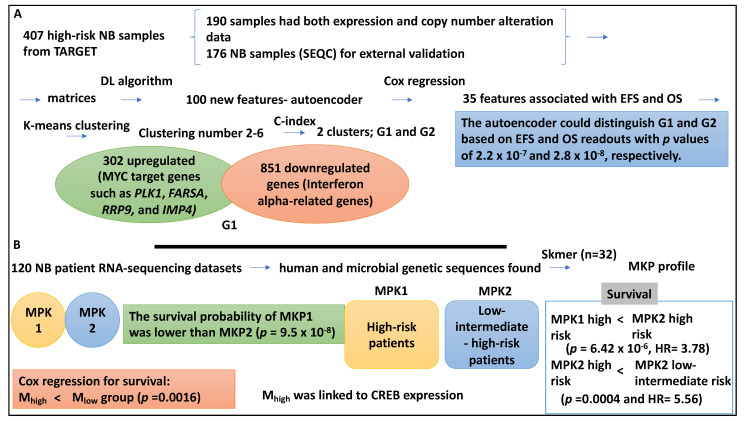
NB patient risk stratification studies informed by ML and associated methods. (**A**) 407 high-risk NB samples from TARGET comprised 217 and 380 gene expression and copy number alteration datasets. 190 samples had both expression and copy number alteration data, and 176 NB samples were used for validation. Stacked matrices were formed based on the expression and copy number data, and 100 new features were obtained from the autoencoder (DL/DNN). Cox regression reduced the 100 new features to 35 features that were linked to EFS and OS (*p* < 0.05). K-means clustering and C-index split the group into G1 and G2. The autoencoder could distinguish G1 and G2 based on EFS and OS readouts with *p* values of 2.2 × 10^−7^ and 2.8 × 10^−8^, respectively. 302 and 851 up- and downregulated genes in the G1 subgroup. For example, MYC targets *PLK1*, *FARSA*, *RRP9*, and *IMP4* were upregulated in the G1 subtype, and interferon alpha-related genes were downregulated in G1. (**B**) 120 NB patient RNA-sequencing datasets were processed for human and microbial sequences. Skmer extracted k-mer patterns (K = 32) of microbiome sequences (MKP profile); therefore, the group was split into MKP1 and MKP2 (the survival probability of MKP1 was lower than that of MKP2). The high-risk patients were represented in both MKP clusters, but the low- and intermediate-risk cases were represented in the MKP2 cluster only. The high-risk cases allocated to the MKP1 group showed lower survival than their counterparts allocated to the MKP2 group (*p* value of 6.422 × 10^−6^, HR = 3.78). High-risk NB patients allocated to MKP2 also had lower survival than the low-intermediate-risk NB patients in MKP2 (*p* value of 0.0004 and HR = 5.56). Cox regression for survival showed that patients with M_high_ had shorter survival compared to the M_low_ group (*p* value of 0.0016). Finally, the M_high_ group showed CREB expression.

The authors used a t-test to distinguish between the two subtypes, and 302 and 851 up- and downregulated genes in the G1 subgroups were studied using gene ontology. It was shown that MYC target genes such as *PLK1*, *FARSA*, *RRP9*, and *IMP4* were upregulated in the G1 subtype (*p* value of 9.81 × 10^−7^) [42]. MYCN amplification was more frequently found in the ultra-high-risk group in association with the overexpression of *MYCN/MYC* genes [42]. Interferon alpha-related genes were represented in the downregulated genes of the G1 subgroup (*p* value of 5.14 × 10^−3^) [42]. In conclusion, the prognostic subtypes were identified by DL (DNN) approaches and validated by other classifiers, showing two distinct groups within the NB high-risk groups with distinct prognoses (Figure 6A).

Interestingly, NB risk groups may also be linked to the tumour intracellular microbiome, and a study aimed to link these two aspects [43]. This study used 120 NB patient RNA-sequencing datasets from the National Cancer Institute for human and microbial genetic sequences. In this cohort, the mean age at diagnosis was four years and three months, and the majority were male and were classified as high-risk based on COG criteria (80.8%) [43]. Microbiota was found in the NB patient RNA-sequencing data by Skmer, which extracted k-mer (K = 32) patterns of microbiome sequences, and this was referred to as the MKP profile; as a result, the group was split into MKP1 and MKP2. The survival probability of MKP1 was lower than that of MKP2 (*p* = 9.5 × 10^−8^). The Pearson Chi-square test also showed that risk groups were linked to MKP groups (*p* = 0.0195). Accordingly, the high-risk patients were represented in both MKP clusters, but the low- and intermediate-risk cases were represented in the MKP2 cluster only [43]. This suggested that the risk group was linked to the MPK profile. As expected, the high-risk cases allocated to the MKP1 group showed lower survival than their counterparts in the MKP2 group (*p* value of 6.422 × 10^−6^, HR = 3.78). High-risk NB patients allocated to MKP2 also had lower survival than the low-intermediate risk NB patients in MKP2 (*p* value of 0.0004 and HR = 5.56) [43]. In addition, it was shown that microbial gene abundance was highly linked to prediction accuracy, and as such, the microbiome prediction score (M-score) was introduced. The M-score split the high-risk patients into two M_high_ and M_Low_ groups with high accuracy compared to the current COG risk stratification. For example, the Cox regression for survival showed that patients with M_high_ had shorter survival and higher clinical risk compared to the M_low_ group (*p* value = 0.0016). Finally, the M_high_ group showed CREB activation, which may induce genes involved in proliferation, angiogenesis, and apoptosis [43]. In conclusion, the intracellular microbiota may impact signals that influence patient survival and inform COG risk stratification (Figure 6B).

In summary, copy number and expression data can be transformed into matrices; these will then be fed to the encoder (DL), followed by Cox regression to reduce features and estimate EFS and OS. K-means clustering and C-index can support deciphering specific groups. The performance of the encoder (DL) can be compared to other methods. Each risk group identified can be further tested with gene ontology. Finally, microbiota in NB expression data could be identified by SKmer analysis, and this defined an MKP profile with distinct survival estimates. Using the Pearson Chi-square test, it is possible to link the risk groups with the MPK profiles. Cox regression could link the microbiome prediction score with survival.

### 3.2. ML to Predict NB Patient Outcomes, including Survival

As mentioned earlier, patient outcomes may encapsulate a range of measurable metrics, including survival, mortality rate, and the like. This section aims to summarise studies that have measured outcomes, including survival. A study used a gene expression-based classification system to predict NB patient outcomes, and these outcomes were linked to tumour hypoxic conditions [44]. Hypoxia, defined as low oxygen levels in tumours, may influence tumour growth, treatment, and cancer stem cells and drive more aggressive tumour behaviour, making this aspect relevant to patient outcomes [44,45,46,47]. This study utilised an ANN (MLP) method to establish an NB patient predictive model linked to tumour hypoxia. The study set up a classifier (ANN), which they refer to as the “NB-hypo classifier”, and a total of 182 patient microarray datasets were obtained from four cohorts (including data from a children’s university hospital in Essen, Germany) and were split into 100 and 82 for training and testing, respectively. The classifier was trained based on a leave-one-out cross-validation strategy, while it was then tested using the 82 testing datasets [44]. Accordingly, for the 82 NB tumour datasets, the classifier predicted 53/59 (90%) good outcomes and 18/23 (78%) poor outcomes; as a result, the accuracy for patient outcome prediction was estimated at 87% [44]. In addition, the performance of the classifier for various pathological parameters, such as MYCN and age, was established. For example, MYCN status displayed the highest sensitivity and the lowest specificity, while age at the point of diagnosis showed an opposite trend, and the INSS stage showed more balanced specificity and sensitivity levels.

**Figure 7:**
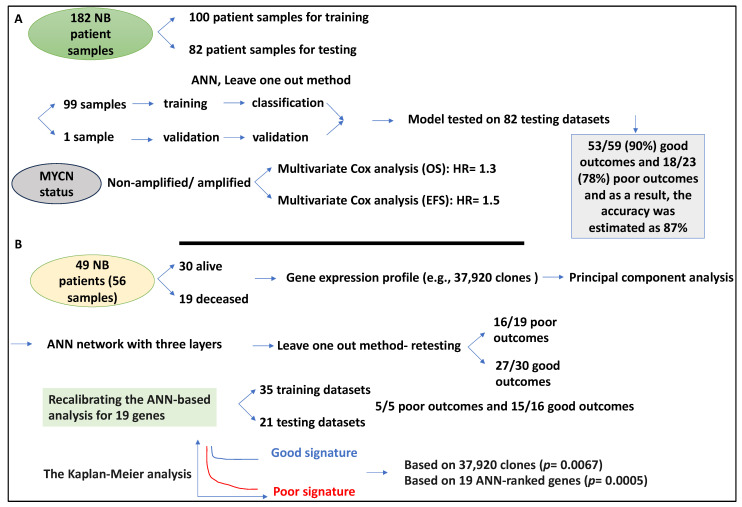
ML to predict NB patient outcomes. (**A**) A study comprised 182 patient microarray datasets split into 100 and 82 training and testing datasets, respectively. This study developed an MLP (ANN) method, and the training dataset underwent sampling and testing using a leave-one-out and retesting method. For example, the 100 datasets were split into 99 and 1 (for training and validation) and the classifier was therefore trained and validated. The model was tested using the 82 testing datasets. This ANN classifier performed well, for example, it predicted 53/59 (90%) good outcomes and 18/23 (78%) poor outcomes, and the accuracy for patient outcome prediction was 87%. (**B**) Another study also used an ANN to predict high-risk NB patient outcomes. Accordingly, 56 NB patient samples (from 49 patients) were split into 30, and 19 alive and deceased groups, respectively. These samples were studied for 37,920 expression clones and were reduced to 10 using principal component analysis. Following this, an ANN model was built. A leave-one-out and retesting strategy was employed and successfully predicted poor and good outcomes in 16/19 and 27/30 patients, respectively. Further, recalibrating the ANN-based analysis (for 19 genes, including *DLK1* and *SLIT3*) and gene minimisation were performed, and 35 and 21 datasets were selected for training and testing, respectively. This classifier predicted 5/5 poor outcomes and 15/16 good outcomes. Kaplan-Meier analysis also revealed that the ANN successfully split the high-risk patients into good and poor prognosis groups based on 37,920 clones (*p* = 0.0067) and 19 ANN-ranked genes (*p* = 0.0005).

NB-hypo classifier, followed by Kaplan–Meier curves and log-rank analyses, split the patients into two groups with good and poor prognoses with distinct OS and EFS values (*p* < 0.0001). For good versus poor distinction, the NB-hypo obtained an HR of 3.3 and 3 for OS and EFS, respectively. INSS and MYCN status were also linked to OS and EFS based on a multivariate Cox analysis. For example, for MYCN normal and amplified status, an HR of 1.3 and 1.5 for OS and EFS were obtained, respectively [44]. The concordance between predicted and actual patient outcomes was 48/49 (98%) in localised disease (stages 1–3) and 4S, therefore, a 2% error rate was detected [44]. In contrast, the most misclassified patients were stage 4. The classifier predicted five low/intermediate-risk patients with 100% accuracy. Gene ontology analyses showed the enrichment of the hypoxia term for the poor outcome group, revealing that prognosis classification was linked to hypoxic states [44]. In conclusion, this model enhanced the NB patient outcome predictions and was useful for early-stage patients (Figure 7A).

In an interesting study from Javed Khan’s group, the authors attempted to use ANN to more accurately predict outcomes for NB patient risk groups, since even though the current risk stratification criteria are thorough, the survival rate of high-risk NB patients remains at less than 30% [48]. To that end, they performed cDNA microarray profiling, comprising clones, to train an ANN to accurately predict survival. Specifically, they collected 56 treatment-naïve primary NB tumours (from 49 NB patients) and patients were split into good and poor outcome groups based on EFS. For example, the former displayed no relapse or progression for at least a total of 36 months (n = 30), while the latter died due to NB (n = 19) and these tissues were studied by microarray. It is also noteworthy to mention that the 56 samples were split into 35 and 21 training and testing groups, respectively [48]. The 56 NB samples were studied for 37,920 clones and processed using principal component analysis, and this analysis split the samples by their outcomes. Of the 37,920 clones selected, 10 passed the principal component analysis, which reduced dimensionality and overfitting. Further, an ANN network with three layers (an input layer with 10 principal component analysis or gene expression data, a hidden layer with 3 nodes, and an output layer providing outcome votes) was generated. Using a leave-one-out strategy, the authors tested the samples during training of the ANN and assessed the prediction of outcomes for all 37,920 clones [48]. Accordingly, this ANN predicted 16/19 poor outcomes and 27/30 good outcome patients, thus bearing a sensitivity and specificity of 84 and 90% for poor and good outcomes, respectively. Further, positive predictive values of 84% and 90% were obtained for poor and good outcomes, respectively [48].

Further, the top 24 ANN-ranked clones (linked to 19 genes, including *DLK1* and *SLIT3*) more robustly separated poor from good outcomes compared to the 37,920 clones; this was performed by gene minimisation methods and principal component analysis [48]. By recalibrating the ANN with the 19-gene signature and 35 training datasets, this classifier predicted 5/5 poor outcomes and 15/16 good outcomes, with a sensitivity and specificity of 100 and 94%, respectively [48]. Finally, the Kaplan–Meier analysis was used for survival prediction and showed that the ANN could partition the high-risk patients based on 37,920 clones (*p* value of 0.0067) and 19 ANN-ranked genes (*p* value of 0.0005) (Figure 7B) [48]. 

In summary, ANN methods with a leave-one-out strategy can be used to test NB expression data, followed by statistical analysis to identify patients with good or poor outcomes and distinct OS and EFS. Samples of patients with poor outcome classifications were linked to hypoxia. Finally, the latter study provided evidence that gene expression clones can be reduced in dimensionality with principal component analysis and then classified by ANN. Kaplan–Meier analysis can predict the survival of patients.

The accurate prediction of survival of NB patients can be viewed as a strategy for refining treatment stratification to minimise overtreatment [49]. ML has been applied to patient survival prediction using algorithms including SVM [19], DNN/DL [20] and RF [22]. Ensemble classifiers enhance prediction performance, and outperform single classifiers such as DT and SVM, and can be homogenous or heterogeneous based on the classification algorithms used [19,49,50].

**Figure 8:**
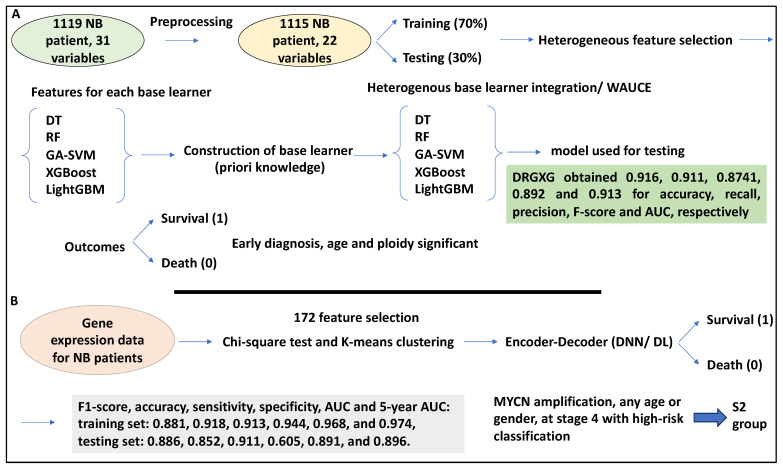
ML to predict NB patient survival. (**A**) 1119 NB patient samples and 31 variables were initially selected, and reprocessing reduced this value to 1115 NB patient samples and 22 variables. After preprocessing, 5 base learners were developed (DT, RF, SVM, XGBoost, and LightGBM). Further, a method was devised to extract optimal features relevant to each base learner and this guided the formation of the base learner (priori knowledge). The heterogeneous base learners were then integrated by WAUCE. DRGXG obtained 0.916, 0.911, 0.8741, 0.892, and 0.913 for accuracy, recall, precision, F-score, and AUC, respectively. The classifier could predict a binary, alive (1) or dead (0) outcome. Early diagnosis, age at diagnosis, and ploidy were all significant. (**B**) Survival in NB patients was predicted using a DL method based on gene expression profiles. 721 NB patient microarray data accompanied by survival and other clinical data, was obtained. Chi-square tests supported feature selection, and 172 features were retrieved. K-means clustering divided the patients and features into S1 and S2 groups. A DL/DNN comprising an encoder (with an attention mechanism) was fed the subgroup features, and the decoder predicted survival (alive/dead status) in NB patients. F1-score, accuracy, sensitivity, specificity, AUC, and 5-year AUC were established for the classifier in this study; for the training set it was 0.881, 0.918, 0.913, 0.944, 0.968, and 0.974 and for the testing, it was 0.886, 0.852, 0.911, 0.605, 0.891, and 0.896, respectively. Patients with MYCN amplification status, of any age, or gender, at stage 4 with a high-risk classification were placed in the S2 group.

In a study, the authors applied a heterogenous ensemble learning method (DRGXG) to assist NB patient survival prediction [49]. This learning method consisted of data preprocessing by selecting 1119 NB patient records with 31 specific variables from TARGET. Preprocessing eliminated datasets with missing values, leaving a total of 1115 samples and 22 variables. Further, these patient datasets received a vs. label to denote the status of survival, alive (−1) or dead (1). By that token, 689 patients were alive, and the remaining were dead [49]. This data was then split 70/30 for training and testing processes [49]. The heterogeneous ensemble learning strategy used was composed of three sections: the heterogenous feature selection (HFS), heterogeneous base learners, and the weighted area under the curve-based base learner integration (WAUCE). Accordingly, after data preprocessing, 5 heterogenous base learners were developed, comprising 5 base learners (DT, RF, SVM, and light and extreme gradient boosting (lightGBM and XGBoost)). Each of these base learners was then processed by an HFS method to retrieve relevant optimal features from each base learner. The effective feature subsets for each base learner guided the formation of the base learners (priori knowledge). Subsequently, the five heterogeneous base learners were integrated by the WAUCE [49]. The resulting method described formed DRGXG, which was also applied to testing datasets [49]. The proposed method (DRGXG) obtained 0.916, 0.911, 0.8741, 0.892, and 0.913 for accuracy, recall, precision, F1-score, and AUC, respectively, performing better than single classifiers. Thus, the proposed method achieved accuracy, recall, and AUC of over 90% [49]. By setting a binary outcome of “survival” and “death” by the proposed method, in NB patients, the earlier the diagnosis, the greater the likelihood that the prediction model would predict a “survival” outcome. Also, age at diagnosis was significant; the older the patient is at diagnosis, the greater the risk of “death” outcome. Also, the larger the ploidy value, the greater the likelihood that the prediction model would assign “survival” as the status of the patient [49]. Finally, 10 rules were extracted from DRGXG to predict the survival status of patients (i.e., “dead” or “alive”) with a higher than 90% accuracy. One example is the year of diagnosis > 2000, year of last follow-up ≤ 2013, INSS state = 4, and grade = 0, classing the patient as 1 (alive) [49]. In conclusion, the proposed method extracted valuable information, performed well, and may be able to assist clinical judgements (Figure 8A).

In another study, the authors utilised DL to accurately predict survival in NB patients based on gene expression. Accordingly, 721 NB patient microarray data, including GSE49710, was utilised and the accompanying clinical information, including patient survival, was also downloaded. Having applied the chi-square test to features and survival in the patient datasets, 172 features were selected. These patients and features were then divided into two groups using K-means clustering, whereby 50 genes marked the S1 group, and 122 genes marked the S2 group. MYCN amplification was associated with the S2 group and the S2 group also showed reduced survival compared to S1 [51]. Further, gene ontology analyses showed that the S1 group was enriched for the JAK/STAT pathway, while the S2 group showed enrichment for bone morphogenesis and migration [51].

Further, in a supervised classification model with attention mechanisms, a DNN (DL) was constructed in which both groups of features were fed to the encoder, while the decoder predicted survival probabilities with binary alive and dead outputs [51]. F1-score, accuracy, sensitivity, specificity, AUC, and 5-year AUC were established for the proposed classifier in this study for the training set as 0.881, 0.918, 0.913, 0.944, 0.968, and 0.974, and for the testing set as 0.886, 0.852, 0.911, 0.605, 0.891, and 0.896, respectively [51]. The proposed classifier in this study (the encoder-decoder) was compared with other classifiers. For example, AUCs of 0.65, 0.43, 0.72, and 0.89 were obtained for the MYCN amplification model, Zhong, De Preter, and the current model, respectively [51].

Clinical relevance analyses were established using multivariate Cox regression, and parameters such as MYCN status, age, risk, and stage were tested, which were significant (*p* > 0.05). The authors also built a nomogram with a calibration curve capable of predicting patient survival. For example, patients with MYCN amplification status, of any age or gender, at stage 4 with a high-risk classification were placed in the S2 group, suggesting high clinical relevance [51]. Overall, this study used a DL-based model using 172 features to class patients into two groups and predict their survival status (Figure 8B).

In summary, heterogeneous ensemble learners can be used for patient survival prediction. These heterogeneous ensemble learners usually comprise an HFS, heterogeneous base learners, and WAUCE. The output of this machine can then be tested using various metrics. Finally, DL can also be used for survival prediction. Based on gene expression data, chi-square and K-mean clustering can select features and partition patients and genes into groups. The groups of features can then be fed to a DL method with an attention mechanism to make survival predictions. This section outlined the prediction of outcomes using ML methods.

### 3.3. ML to Predict NB Response to Treatment

Predicting response to treatment in NB patients can be of significant clinical value [27]. A study attempted to use a CNN method to analyse diagnostic metaiodobenzylguanidine (MIBG) scans and thereby predict response to chemotherapy in NB. Accordingly, MIBG scans utilise radionucleotides to image patients, and this imaging then contributes to the Curie score for the disease burden assessment [27]. Despite this, no method can currently predict responses to induction chemotherapy and the related Curie score ≤ 2 at the time of diagnosis. This study used MIBG scans available in the INRG database of patients registered with the Children’s Oncology Group trial, ANBL12P1. Of a total of 146 eligible patients, 43 were excluded due to missing diagnoses or follow-up scans, or scans were improperly formatted, and a total of 103 patients were selected. The patients with a Curie score ≤ 2 responded to four cycles of induction chemotherapy (n = 67), whereas the nonresponders (n = 36) displayed a Curie score >2 and were more likely to have advanced INSS stage of disease and non-MYCN amplification status. As such, 82% of responders were MYCN-amplified, and 56% of non-MYCN-amplified patients were responders [27].

From the viewpoint of the tools used, the authors applied a DL method (CNN). In this case, diagnostic MIBG scans were fed as whole-body 2D images, and the response to induction chemotherapy was predicted by CNN. In addition, a clinical classification logistic regression used age, stage, and MYCN amplification status to predict response to four cycles of induction chemotherapy. Also, a naïve Bayes ensemble classifier was used to analyse the probabilities obtained from the CNN and clinical model to predict patient outcomes. Overall, the AUCs for the CNN, clinical, naïve Bayes, and geometric models were used and their performance was calculated [27].

Class-activation heatmaps were formed using the Grad-CAM method, and this showed that CNN used disease areas of the scans to produce prediction outputs. For example, an original MIBG image was given, and the same image superimposed with the grad-CAM attention-based heatmap obtained from the CNN analysis was also shown. This composite utilised read colours for the areas that CNN paid the most attention to and was therefore clinically important [27]. The performance of the CNN, clinical classification, naïve Bayes, and the combined models (geometric mean) were assessed as AUC: 0.63, 0.65, 0.67, and 0.73, respectively. Overall, the geometric means combined model was the highest performer [27].

**Figure 9:**
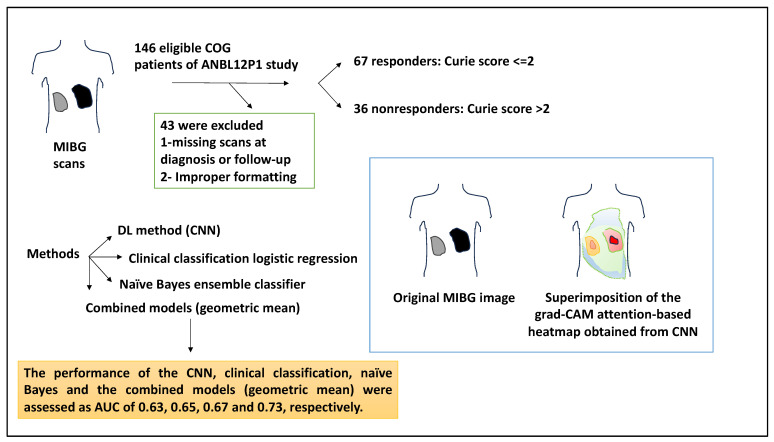
Predicting response to treatment in NB patients based on MIBG scans and ML. A study used the information of NB patients from COG represented in the ANBL12P1 trial. Of 146 eligible patients, 43 were eliminated due to missing scans at diagnosis or follow-up or improper formatting. Of the remaining 103 patients included in this study, 67 were responders and had a Curie score of ≤2, while the nonresponders had a Curie core of >2 (36). 4 models were used in this study, including a DL method (CNN), clinical classification logistic regression, naïve Bayes ensemble classifier, and a combined model (geometric means). The performance of CNN, clinical classification, naïve Bayes and the combined geometric method were assessed as having an AUC of 0.63, 0.65, 0.67, and 0.73, respectively. An original MIBG image and the same image superimposed with the grad-CAM attention-based heatmap obtained from the CNN analysis was given. This composite utilised read colours for the areas that CNN paid attention to.

To understand why the combined model performed better, the errors in each model were analysed. For example, of the 45 incorrect CNN predictions, 55% were predicted accurately by the clinical model. The clinical model had a higher accuracy than CNN (66% compared to 56%), and this may have been because the clinical model predicted that patients with localised disease or MYCN amplification would respond to chemotherapy. On the other hand, the clinical model without CNN was also insufficient since CNN could rely on features other than MYCN and stage to make predictions, and of the 35 patients predicted incorrectly by the clinical model, 15 (43%) were accurately predicted by CNN [27] (Figure 9). In summary, the authors demonstrated that ML can be used to make MIBG diagnostic scans to predict responses to chemotherapy. The studies catalogued in this work have been summarised in Table 1.

## 4. Discussion

Neuroblastoma, a paediatric malignancy of the peripheral nervous system, is the most common solid malignancy in this age group, except for the malignancies of the cranium [1]. In NB, risk groups are defined based on various parameters, including age, stage, and MYCN amplification [7].

### 4.1. Multi-Omics Data and Relevant Tools for Predicting NB Clinical Aspects

We discussed the utility of approaches to process patient multi-omics data, such as transcriptomics, to predict NB subgroups, risk groups, and INSS staging [28,32,33]. Similarly, immune-metabolism-linked gene expression similar to MYCN amplification status can be linked to prognosis in NB. Initially, differentially expressed immune genes were processed with R software (v4.2.1) and ML [52]. This then led to the development of risk scores. Based on these models, NB patients were grouped according to their prognostic scores. Additionally, 89 immune-metabolism genes were differentially expressed between MYCN-amplified and non-MYCN-amplified states. A subset including *GLDC*, *GNAL1*, *ABCC4*, and *CNR1* was chosen by ML to generate a prognostic model [52]. The Kaplan–Meier curve and 5-year AUC-ROC showed that the model could predict patient prognosis, and this was linked to expression levels of immune-related genes [52].

From the viewpoint of the methods used for subtype and INSS stage discrimination and network building, several points can be made. For subtype stratification, transcriptomics followed by principal component analysis and unsupervised hierarchical clustering in NB can be utilised. This approach may also inform specific targets for therapy and the establishment of personalised therapies (for example, four subtypes, including one new subtype identified in NB, were predicated on a 6-gene expression profile that corresponded to previously postulated subtypes) [32]. Although these alternative classification systems are very important, more work is required for their thorough characterisation and implementation in routine clinical NB diagnostics. In addition, using qPCR, univariate Cox regression, and principal component analysis, a one-score Y96 NB risk predictor model could be generated to reproducibly and easily classify NB risk groups [33]. Although this model is well-developed and practical and could complement the current risk stratification systems routinely used in clinics, more testing in large and independent cohorts is required for a better validation of this predictor. Both studies relied heavily on principle component analysis, which may suffer from pitfalls such as sensitivity to outliers and the requirement for the components to be distinct, which otherwise may lead to random and spurious results [53]. Finally, using ML for INSS staging was discussed earlier. Accordingly, DNN can be applied to expression data from GEO and TCGA for patient staging predictions, by which the DNN architecture was fed with INSS stage and gene expression information matrices. This method yielded a reasonably high AUC and OVRs for the training but not for the testing dataset. Therefore, the pitfall of the approach might be the overfitting of stages 1, 2, and 4S or the lack of distinct enough features between these stages. The implementation of this approach in clinics also needs more reliable results and reproducibility [28]. In conclusion, prognosis in NB is linked to MYCN status, and various expression datasets, including immune-gene expression [52], can be linked to prognoses, and ML and statistical tools were effective in deciphering these links.

Next, methylome data analysed by ML was discussed [37,38]. From the viewpoint of the methods used for analysing methylome data by ML for making clinically relevant predictions, some points can be made. The link between MYCN amplification and CpG islands can be investigated using RFE and ML. For example, of the 126 NB samples, 369,065 CpGs were obtained, and the list was narrowed to 663 differentially methylated CpGs by the ChAMP programme. In total, 25/663 CpG features selected by RFE were used to build the SVM ML model, which showed 100% accuracy for the classification of NB patients based on MYCN status. Finally, Kaplan–Meier survival plots and Cox regression were used to link CpGs with patient survival and MYCN amplification status [37]. Since the prediction accuracy was high, this showed the strength of the model, and as such, DNA-methylation-based diagnoses have been applied to clinics for meningioma [54]. Despite these strengths, the pitfall is that such studies are purely computational, and RFE can also be affected by higher dimensionality. The results of the study need future functional studies for implementation in routine clinical practice [37].

Finally, analysing 493 NB methylome data (Human Methylation 450 K dataset) with principal component analysis yielded four clusters of patients based on MYCN amplification status, and the adequacy and accuracy of the approach for each of the four clusters (subgroups) were established using RF. Feature selection based on probe annotation may also be relevant, and enhancer probes may display a higher level of accuracy compared to other loci of the gene model [38]. Overall, DNA methylation can be accurately analysed by SVM and RF to predict patient clinical aspects [38].

An interesting paper attempted to address one main issue of working with multi-omics data and generating networks, which is the heterogeneity and differences in the data dimensionality [55]. They suggested using a two-network-based approach to integrate these data for NB and called this approach the patient similarity network. The initial step in setting up this network was computing distances within individual patients from specific omics features. Also, the fusion of different omics datasets could be envisaged in two ways: network-level fusion and feature-level fusion. The former was achieved by similarity network fusion algorithms by merging the patient-similar networks derived for each multi-omics dataset, and the latter was obtained by the fusion of features obtained from individual patient similarity networks. Accordingly, the authors used two high-risk NB datasets from SEQC and TARGET and applied the DNN and ML methods with RFE. Finally, for the integration of omics data, network-level fusion worked better [55]. Such approaches are relevant to complex diseases such as cancer, since network-level integration may alleviate pitfalls such as small sample size, heterogeneity, and high dimensionality [55]. In conclusion, networks of multi-omics data could handle the heterogeneity and dimensionality of large datasets.

### 4.2. Histological Data and Relevant Tools for Predicting Clinical Aspects

Histological data that may be utilised by ML for patient prognosis predictions was also discussed in this review [17,39,41]. Similarly, DL (DNN) approaches were proposed by Gheisari et al. to classify digital images of NB into five groups [56]. Accordingly, they processed the input images by whitening methods, and these data were divided into mini-batches and fed to the input layer of the three-layer convolutional deep belief network. This convolutional deep belief network then extracted features from the images and fed them to the encoding block (bag of features as a feature encoding tool) that enabled distinct feature yield, followed by classification using an SVM classifier [56]. The data used for this purpose comprised 1043 NB histological images obtained from the Aperio ScanScope system, representing 125 patients from various neuroblastic tumour classes (including differentiating and undifferentiated NB, poorly differentiated NB, ganglioneuroma, and ganglioneuroblastoma). Accordingly, images were cropped to 300 × 300 pixels and were large enough to encompass the diagnostic feature per neuroblastic type. The proposed model (i.e., a convoluted deep belief network, a bag of features, and SVM) gained precision, recall, and F-measure of 82.54, 85.63, and 84.02%, respectively, by utilising high-level features, and this setup performed better than other methods. In conclusion, they proposed that the method was effective in histological image classification for NB [56].

Given the methods used for histological grading and relevant predictions, a classifier (including SVM) would be trained on features extracted from slides, and then the trained classifier would be tested on the testing dataset. One of the most important aspects of such studies is correct feature (input) extraction, and future work might aim at improving higher-order decision information to improve global labelling and configuration. The expansion of feature groups and the improvement in training dataset collection will all impact the performance of the overall system [17]. The use of SIFT can assist in the retrieval of distinct features since it is robust to scale variation from images while using the bag of features can reduce the number of features [39]. Having extracted distinct features with SIFT and fed them to the classifier (SVM) and the bag of features, the samples can be classed, for instance, into five classes of neuroblastic subtypes, and good precision, recall, and F-measure can be obtained. The advantage of the proposed method is that it is robust to scale variations, and combining it with the bag of features can improve classification accuracy. One of the pitfalls of the classification of NB histology images is the lack of publicly available data, which severely limits the number of samples included in each study [39]. Also, SIFT did not give 100% specificity and the extracted features with SIFT were vectors; as such, it remains to be determined if mathematical features always represent biological features with clinical sequelae. Also, SVM might suffer from long learning times and issues with model interpretation [39].

In another study discussed earlier, nuclear instance segmentation, feature extraction, and per-patient feature aggregation were used for feature retrieval, and this was then followed by feature reduction, selection, and model construction (ML) and good accuracy was obtained [41]. As mentioned, one of the limitations of most NB studies is the low number of samples; further, nuclei segmentation processes may require fine-tuning. One solution is better labelling of the NB dataset, and therefore improving both input and training processes. Overall, these extracted nuclear features may complement the INPC diagnostic module and further assist pathologists [41].

Finally, other methods can also be used for histological classification in NB. Input data of mini-batch scale can be fed to a convolutional deep belief network and its encoding block (containing a bag of features) can yield distinct features, and finally, SVM can be used for classification [56]. As a common problem with all NB studies, the current study also suffered from low sample NB, which limited its clinical implementation [56].

Other studies in NB histology have also used ML for the quantification of immune cell content within the H&E slides. Accordingly, EUNet, a DL/DNN tool with an efficient encoder, was trained to identify lymphocytes in slides stained with CD3 (a marker of T cells). The training set included 3782 images (tiles) obtained from 54 NB whole slides and within this dataset. In total, 73,751 lymphocytes were manually annotated and formed the NeSTBG database [57]. The indicated tiles were used as a training dataset for DL (DNN) (EUNet, comprising an encoder and decoder module) to generate density maps. The decoder contained three layers, the feature map, the up-sampled feature map, and the concatenated feature map [57], while the encoder also used efficientNet-B3 to output predicted density maps. These maps from different layers of the NN at various stages of training were then processed via topological data analysis (TDA). One of the features of TDA was the uniform manifold approximation and projection (UMAP) to reduce dimensionality and perform hierarchical clustering. This setup yielded good results with an absolute error of 3.1 for the testing dataset. In addition, the concordance between the lymphocyte densities predicted and expertly estimated by pathologists was high [57]. The novelty of the system was the use of DL (DNN) to predict density maps, which were aligned with the pathologist’s reports and estimates. This was one of the first attempts to utilise artificial intelligence for the processing of whole slide images from the viewpoint of CD3 T cells, but this can be extended to other types of immune cells [57]. Future work might be aimed at the development of tools to rapidly quantify immune cells in tumour samples to better support pathologists in the clinical decision-making process [58]. Despite this potential, the current study requires deepening to cover other markers (with clinical relevance) such as the presence of PD-1 and PD-L1 (immune checkpoints and their ligands) to correlate immune cell infiltration with the expression of these markers in NB tumours [57].

### 4.3. Medical Imaging Data and Relevant Tools for Predicting NB Clinical Aspects

CT scans linked to ML can be utilised for predicting MYCN-amplified NB [18]. Another study aimed to associate clinicopathological parameters and CT-scan radiographic features to construct a model to predict MYCN status. In total, 172 patients were selected with MYCN-amplified (n = 47) and MYCN-non-amplified (n = 125) status [59]. This cohort was split into training and testing datasets. The clinical model was built based on MYCN amplification status, INSS stage, and Shimida classification among other criteria. Consistently, the authors extracted first, second, and third-order features from regions of interest retrieved from 3-phrase CT images [59]. Dimensionality was reduced using tools such as LASSO and mRMR. For example, 1218 radiomic features were retrieved from the region of interest, and these were reduced to 14 optimal features (1 first-order feature, 5 log-transformed, and 8 wavelets transformed) and were used to construct a radiomic model. Features of the training and testing groups were selected and used by ML tools such as logistic regression, SVM, RF, and Bayes, and their performance was reported in the AUC-ROC values [59]. For example, the AUC for logistics, SVM, Bayes, and RF, were 0.94, 0.94, 0.78, and 0.92 for the training group, respectively. These numbers were 0.909, 0.909, 0.729, and 0.85 for the testing group, respectively. Therefore, the logistic, SVM, and RF classifiers performed better than the Bayes model (*p* < 0.005). Finally, a nomogram comprising data on clinicopathologic aspects and radiomics features was formed using multivariate logistic regression. The nomogram performed better than the clinical model alone (0.77 and 0.946 for training, and 0.917 and 0.977 for training datasets, respectively). The logistic radiomics model performed similarly to the nomogram [59].

From the viewpoint of the methods used for CT scan data analysis reviewed in this article, ANN methods performed better than other algorithms (2D-CNN, Lasso and elastic-net regression, RF, and SVM) for predicting mortality, metastasis, IDRF, MKI, and MYCN status, except for the grade of differentiation from CT scans. In addition, it is possible to use nested cross-validation methods to split training and testing datasets repeatedly to prevent overfitting [18]. Also, there is a difference between 2D and 3D classifiers. The 2D-CNN classification method performed weakly due to the small data size, while the 3D radiomics methods did not suffer from this issue since these models have access to NB statistical information in a higher dimension compared to the 2D-CNN model. In addition, heterogeneity and training on slices that were not related to the outcome may have affected 2D-CNN. CNN is also data-hungry, and the small size of the study could have been a factor [18]. In addition, another important point may be using multiple ML methods may be a positive aspect of analysing CT scans to form a more robust final prediction. Due to the limited sample size, future work could focus on acquiring more samples and also automating the processes to analyse large-scale datasets [18]. It is possible to use clinicopathological parameters and radiographic features (from CT scans) to develop predictive models. Dimensions can be reduced by LASSO and mRMR. The selected features from the training datasets can be used to develop a radiomic model using SVM, RF, or logistic regression, and performance can be tested. The combined radiographic and clinical features can be processed by multivariate logistic regression to develop a useful nomogram for clinical practice [59]. MRI-based NB clinical aspect classification has also been reported, which is beyond the scope of this work [60,61].

### 4.4. Investigating Clinicopathological Aspects of NB Patients

In addition, in this study, three main aspects of the clinicopathological characteristics of patients that may be investigated using ML, including risk, patient outcome including survival, and treatment, were discussed.

Risk groups and their survival were addressed in this study [42,43]. Similarly, gene expression profiles could be used to form prognostic indicators of high-risk groups to allow for better stratification for treatment purposes. Accordingly, RNA-sequencing data from UCSC Xena were downloaded, and high-risk survival was split into short (n = 22), and long (n = 12) survivals (training data). In total, 40 genes linked to survival prediction for high-risk groups were differentially expressed between short and long-survival groups and included *HOXD10*, *NHLH2*, and *EVX2*. Further, the ML methods used to classify high-risk patients based on the test dataset (GSE49711) for SVM and ANN models were 79% and 82% accurate, respectively [62].

For risk stratification and the useful methods linked to analysing this aspect, some points can be made. For example, high-risk subtype matrices (of expression and copy number) can be transformed into new features. The new features will then be fed to an encoder (for example, DL/DNN algorithms). Cox regression can reduce the features and link them to EFS and OS, while K-mean clustering analysis is complemented by C-index analysis to decipher subgroups. Based on this, high-risk subgroups with distinct EFS and OS can be identified. The autoencoder method could be compared with other methods such as iCluster and principal component analysis [42]. Other ML methods, including SVM, naïve Bayes, logistic regression, and Xboost can also be used. Overall, DL (DNN) could significantly improve the high-risk classification method, and the implementation of this method would reduce over- or undertreatment of high-risk NB cases [42]. In addition, this was one of the studies using DL (DNN) for personalised medicine, specifically risk stratification. Despite this, DL can suffer from issues such as large data requirements and computing power and issues with interpretation and overfitting [63].

Moreover, microbiota in NB expression data could be identified by SKmer and this defined an MKP profile with distinct survival profiles. Using the Pearson Chi-square test and Cox regression, it is possible to link the risk group with the MPK profiles and microbiome prediction score with survival, respectively [43]. ML can be used at the interface of microbiome and genetic risk and can improve diagnosis and prognosis in NB and support more effective risk stratification. In conclusion, ML (DL/DNN, SVM, and ANN) could be used for the better stratification of high-risk patients with divergent survival patterns [62].

NB patient outcomes, including survival and disease recurrence, can be predicted using high-throughput omics data [44,48,64].

From the viewpoint of methods used for outcome analysis, ANN as a classifier can be used to analyse NB expression data. For example, training data may be analysed by a leave-one-out strategy, and the classifier may split the patients into good and poor outcomes. The performance of classifiers for various clinicopathological aspects can be determined. Classification followed by Kaplan-Meier curves and log-rank tests can split the patients by prognosis, and the OS and EFS of each group can be determined, while the concordance between predicted and actual risk and staging groups can also be established [44]. The extrapolation of this study requires larger cohorts to increase confidence in the classification. In addition, adding other molecular angles, such as non-coding RNA and protein data, can further enrich the classification model [44]. In agreement with the previous study, cDNA microarray data can be used to train an ANN to predict the outcomes of patients. For example, in the NB samples processed by microarray, a large number of clones can be reduced to a much smaller number by principal component analyses, and this can then be fed to an ANN network to develop a classification model. The predicting model can split the patients by outcomes with specific sensitivity and specificity, and Kaplan-Meier analysis can express individual survival signatures [48]. The use of larger, prospective clinical trials could improve confidence in this method and allow physicians to tailor individualised therapy based on these predictions. It is noteworthy that ANN can suffer from computational intensiveness and overfitting issues [48].

In addition, other ML tools, such as DNN can be used for NB outcome predictions. DNN has displayed excellent performance with various datasets and parameters, but some issues, including small numbers of samples and a large number of features, remain. DNN can tackle this issue by feature selection and inducing constraints during the learning step. A study used 4 NB tumour datasets, and the DNN showed an accuracy of 85–87%, while SVM and RF showed accuracies of 75–82% [64]. For the clinical outcome of death due to disease and disease progression, DNN obtained balanced accuracies of 87.3% and 84.7%, respectively [64] suggesting DNN can be useful for outcome predictions.

In the survival section, ML algorithms used for constructing models to predict patient survival were addressed [13,49,51]. Another study attempted to combine RF- and ANN-based models to link NB patient genomic data with patient survival [13]. Accordingly, the authors selected the GSE49710 and GSE73517 datasets for training and testing, respectively. GSE49710 featured 176 and 322 high- and low-risk cases, respectively, while GSE73517 featured 56 and 49 high- and low-risk cases, respectively [13]. Initially, the differentially expressed genes in the datasets were determined, and 94 differentially expressed genes were fed to RF. Of the 500 DTs constructed, 290 with the least error were selected, and 32 differentially expressed genes (variables) with an importance of >2 important values were obtained (including, *PLCD4*, *NTRK1*, and *EPS8L1*) [13]. Finally, this subset of 32 genes (variables) was shown to represent the NB high-risk feature genes by the K-means supervised clustering [13]. ANN then constructed a model that showed a high AUC when tested for both the training (0.998) and testing (0.858) datasets. Kaplan–Meier plots also showed a greater OS and PFS for NB patients with low-risk stratification compared to their high-risk counterparts (HR = 3.86 and 3.03, respectively) [13].

From the viewpoint of the methods used for survival predictions, multiple studies were reviewed earlier [13,49,51]. The heterogeneous base learners can be developed, followed by retrieving optimal features for each based learner [49]. These effective feature subsets formed a priori knowledge for the base learners for ensemble construction, and finally, the heterogeneous learners can be integrated and various metrics can be calculated. Such a classifier can then predict outcomes (including survival and death) and the effect of clinicopathological aspects on the risk of each binary outcome [49]. The limitations of such studies could be the dataset size and incomplete patient data. The pros of this method include the higher performance and generalisability of the ensemble classifiers. Future efforts in this field may include dataset retrieval with coordinated molecular and patient record information, larger data size, and the application of this method to other paediatric cancers [49]. Finally, DL (DNN) can be used to predict patient survival. Accordingly, expression datasets can be processed, and features can be selected by chi-square test. K-means clustering can then partition the patients and features into relevant groups. The groups of features can then be fed to a DNN encoder with an attention mechanism to predict a binary alive or dead output and various metrics can be calculated. Links to clinical parameters can then be conducted by multivariate Cox regression, and nomograms can be built to facilitate clinical decision-making [51]. The pitfalls of using DL (DNN) for this purpose can be that DNN may be uninterpretable, and to alleviate this, the authors used attention mechanisms to understand the role of genes in NB. A larger dataset would enhance confidence in the performance of the methods used. Finally, RF and ANN-based models may be useful for survival prediction in the NB [13].

Finally, ML can establish a personalised response to treatment [27,65]. From the viewpoint of the methods used to predict responses to treatment, 4 models, inclusive of CNN, clinical classification, logistic regression, naïve Bayes ensemble classifier, and geometric mean models can be utilised to predict MIBG-informed treatment responses [27]. To enhance the applicability of these methods, it is important to choose larger prospective cohorts. Larger cohorts may also mean that external validation may be possible, multiple drug response time points and conditions can be incorporated into the study, and finally, images and their processing can be standardised [27]. Other methods have also been used. Lombardo and colleagues formed a computational network model based on intracellular pathways involved in NB progression to predict response to PD-L1 treatment. Accordingly, the study simulated the impact of the mentioned intracellular signalling pathways by developing an integrated network of protein kinases and their associated cascades [65]. This model was termed an “ordinary differential equation”. Some interesting observations were made using this model. For example, PD-L1 expression was linked to ALK and ERK activation and AKT inhibition. Further, the model looked at the effect of ALK mutation status on PD-L1 levels. Also, in an ALKF1174L-mutated tumour, the levels of PD-L1 increased. Similarly, the levels of PD-L1 differed when ALK inhibitors were used, and the predicted peak of PD-L1 was much lower when crizotinib (an ALK inhibitor) was administered. This was also seen using COPASI software (version 4.25) to validate the findings using the GSE107354 dataset, whereby the use of crizotinib led to a 4.33-fold decrease in PD-L1 expression. The computational tools mentioned were useful for the therapeutic management of NB patients and were one of the first steps towards a decision support system for the clinical management of patients [65].

Finally, single-cell and protein biology assayed by flow cytometry can also be linked to ML in NB. For example, a study found that using ML, it is possible to provide tomographic imaging and 3D phase-contrast tomograms on single NB cells separated by flow cytometry [66], a promising method for easier detection of NB.

In this study, the use of patient data (including multi-omics, histology, and medical imaging) for ML to predict various NB patient clinical attributes was catalogued. Further, predicting risk groups and outcomes, including survival and treatment, using ML was catalogued. A robust and closer link between ML methods and clinical pursuits may substantially improve clinical decision-making, treatment, and patient outcomes [67].

## Figures and Tables

**Table 1:** Summary of data type and tools used by the main studies and the findings.

Data Type Used and Lab Method	Tools Used	Findings	Reference
59-gene signature obtained from data mining from 579 patient datasets (30 training, 313 testing, 236 validation), and qPCR	Data mining, multivariate Cox regression	Signature predictor of outcome; for example, patients with a higher risk signature, deemed at higher risk of death and relapse with an odds ratio for OS and PFS of 19.32 and 3.96.	[15]
4 × 44 K microarray data from 709 NB specimens	SVM, Kaplan–Meier and Multivariate Cox regression	Classifiers showed the highest clinical value for low- and intermediate-risk patients (low-risk: EFS: 0.84, OS: 0.99, and intermediate-risk, EFS: 0.88, and OS: 1 for these groups.	[23]
47 microarray samples (dataset 1 comprising 23 NB tumours, and dataset 2 comprising 30 NB tumours) and 101 NB samples for validation	Principal component analysis and unsupervised hierarchical clustering	6-gene signature (*ALK*, *BIRC5*, *MYCN*, *CCND1*, *NTRK1*, and *PHOX2B*) identified 4 subgroups (p1–p4). Groups p1–p3 corresponded to subtypes 1,2A, and 2B, but p4 was novel (11q deletion, MYCN-non-amplified, low expression of *ALK*, *BIRC5*, and *PHOX2B* linked to poor outcomes).	[32]
96 samples and tested on 362 separate microarray expression datasets, and RT-PCR	Univariate Cox regression and principal component analysis	A 3-gene (*CDH5*, *PAFAH1B1*, and *NME1*) expression signature for risk stratification, the Y36 predictor model could distinguish 2 groups in OS and EFS (HR, 9.3 and 3.1, respectively), Y96 was also formed. From the 352 validation samples, 2 groups with distinct OS and EFS were distinguished.	[33]
280 NB datasets deposited in GSE85047 and its clinical data were obtained; matrices contained patient data (INSS stage and gene expression array)	DNN architecture	The OVR AUCs for patient stages ranging from 1–4S were 0.8, 0.66, 0.59, 0.85, and 0.58, respectively.	[28]
126 NB samples inclusive of 45 MYCN-amplified and 81 non-MYCN-amplified datasets and 663 differentially methylated CpGs were obtained	ChAMP programme, RFE, and ML (SVM), hierarchical and K-means clustering, Kaplan–Meier and Cox regression	14 genes, including *NXPH1* and *SOX2-OT* were highly methylated. 25/663 of these islands and the 663 CpGs led to correct clustering based on MYCN status. MYCN amplification status was associated with CpG score and patient survival (OS: HR = 5.11, EFS: HR = 4.84).	[37]
493 NB methylome data referred to as the Human Methylation 450 K dataset	Principal component analysis and RF	Clustering based on MYCN led to 4 clusters: A, MYCN-amplified patients; B, stage 4 INSS without MYCN amplification; C, stage INSS 4 patients; and D, stage I-III without MYCN amplification. RF made accurate classifications for groups A and B (these groups were linked to DNA methylome). MYCN-amplified A group was linked to DNA methylation of the enhancer regions.	[38]
Training dataset consisted of 387 cropped image tiles obtained from 3 whole slides	Feature construction, selection, and extraction, classification by SVM, and leave-one-out method	Feature extraction followed by classification by SVM for resolution levels 1, 2, 3 and 4, yielded 3, 6, 10, and 5 features, respectively. Neuroblastic types comprising 10 undifferentiated, 13 differentiated, and 10 poorly differentiated NB, yielded accuracies of 90, 84.62 and 90%, respectively.	[17]
6 TMA slides and 7 whole sections for 125 NB patients (3 datasets in total, 623 for training, 211 for the second section of the validation process, and 209 for testing). 623 sub-images and 5 whole sections for validation	SIFT with the bag of features and the SVM classifier	SIFT with the bag of features and the SVM classifier outperformed other methods with precision, recall, and F-measure were 83.81, 86.61, and 85.19, respectively. The algorithm first assigned a label and then classified using a majority vote. A tissue section (4905) was ganglioneuroma, and 10/10 sub-images were assigned correctly.	[39]
563 H&E whole-slides were obtained from 107 NB patients with two distinct groups of favourable (67) and unfavourable (40) prognoses	Segmentation, feature extraction and per-patient feature aggregation for feature extraction. Feature reduction, feature selection and model construction for ML method	After nuclear instance segmentation, the number of nuclei, those nuclei identified by the algorithm, and false positives were established as 3408, 3407, and 46, respectively, and, a recall and precision of 98.62% and 98.65%, respectively. In both the training and testing datasets, clinicopathological factors such as nucleus morphology intensity of features, and age could accurately classify the patients with an AUC of 0.946.	[41]
3D CT scans of 65 NB patients, primary tumours were segmented from CT scans and reviewed by a radiologist. A pyradiomics library was utilised to retrieve 105 radiomic features	Lasso, logistic and elastic-net regression, ANN, RF and SVM classifiers and 2D CNN	ANN obtained AUC-ROC of 0.79 (0.045) for mortality, 0.83 (0.043) for metastasis. 0.76 (0.021) for IDRF, 0.66 (0.031) for MKI index, and 0.77 (0.038) for MYCN status. For neuroblastic differentiation grade, elastic-net regression obtained and AUC of 0.82 (0.044).	[18]
407 high-risk NB samples were collected from TARGET comprising 217 and 380 gene expression and copy number alteration datasets, 176 NB datasets for validation	DL (DNN), Cox regression, K-means clustering analysis, SVM, naïve Bayes, logistic regression and XGBoost	The autoencoder could distinguish G1 and G2 based on EFS and OS readouts with *p* values of 2.2 × 10^−7^ and 2.8 × 10^−8^, respectively. SVM performed better than the other three classifiers (average AUC of 0.844) and was also able to split the high-risk cases into two subgroups. MYC target genes such as *PLK1*, *FARSA*, *RRP9*, and *IMP4* were upregulated in the G1 subtype (*p* value of 9.81 × 10^−7^).	[42]
120 NB patient RNA-sequencing datasets, the mean age at diagnosis was four years and 3 months, and the majority were male and were classified as high-risk	Skmer, Pearson Chi-square test, Cox regression	Microbiome sequences; MKP1/2 profiles. The survival probability of MKP1 was lower than that of MKP2 (*p* = 9.5 × 10^−8^). The high-risk cases in the MKP1 group showed lower survival than their counterparts in MKP2 group (*p* = 6.422 × 10^−6^, HR = 3.78). High-risk NB patients in MKP2 also had lower survival than the low-intermediate-risk NB patients in MKP2 (*p* = 0.0004 and HR = 5.56).	[43]
182 patient microarray datasets obtained from 4 cohorts (100 training and 82 testing)	ANN and leave-one-out method, Kaplan–Meier plots and log-rank tests	NB-hypo classifiers split the patients based on good and poor prognosis with distinct OS and EFS values (*p*-Value < 0.0001). Errors occurred when classifying stages 1–4 (including 4S), while 100% accuracy was obtained when processing low-intermediate-risk patients.	[44]
56 treatment-naïve primary NB tumours (from 49 patients), 30 alive and 19 (dead), 37,920 clones selected	Principal component analysis, ANN, leave-one-out method, Kaplan–Meier analysis	The ANN could partition the high-risk patients based on 37,920 clones (*p* value of 0.0067) and 19 ANN-ranked genes (*p* value of 0.0005).	[48]
1119 NB patient records with 31 variables, after preprocessing 1115 samples and 22 variables were selected. 689 patients were alive and the remaining were dead	DRGXG: heterogeneous ensemble learning strategy: HFS, heterogeneous base learners (DT, RF, SVM, and lightGBM, and XGBoost) and WAUCE	The earlier the diagnosis, the greater the likelihood that the prediction model would predict a “survival” outcome. The older the patient is at diagnosis, the greater the risk of “death” outcome. The larger the ploidy value, the greater the likelihood that the prediction model would assign “survival” as the status of the patient.	[49]
721 NB patient microarray data, 172 features selected	Chi-square test, K-means clustering, supervised classification model (DNN) with attention mechanism	50 genes marked the S1 group, and 122 genes marked the S2 group. MYCN amplification was associated with the S2 group. Multivariate Cox regression showed significance for MYCN status, age, risk, and stage (*p* > 0.05). Patients with MYCN amplification status, of any age, or gender, at stage 4 with high-risk classification were placed in the S2 group.	[51]
103 patients: patients with a Curie score ≤ 2 responded to induction chemotherapy (n = 67), whereas the nonresponders (n = 36) displayed a Curie score > 2	DL (DNN) method (CNN), clinical classification logistic regression, naïve Bayes ensemble learner/classifier, and geometric models, Grad-CAM method	An original MIBG image and the same image superimposed with the grad-CAM attention-based heatmap obtained from the CNN analysis were shown. This composite utilised read colours for the areas that CNN paid the most attention to. The performance of the CNN, clinical classification, naïve Bayes, and the combined models (geometric mean) were AUC: 0.63, 0.65, 0.67, and 0.73, respectively.	[27]

## Data Availability

Not applicable.

## Abbreviations

ALT	Alternating length of telomerase
ANN	Artificial neural network
CAD	Computer-aided diagnostics
CIMP	CpG island methylator phenotype
CNN	Convolutional neural network
DL	Deep learning
DNN	Deep neural network
EFS	Event-free survival
GEO	Gene expression omnibus
HFS	Heterogenous feature selection
HR	Hazard ratio
IDRF	Image-defined risk factors
INPC	International neuroblastoma pathology classification
INSS	International neuroblastoma staging system
INRGSS	International neuroblastoma risk group staging system
MIBG	Metaiodobenzylguanidine
MKI	Mitosis-karyorrhexis index
ML	Machine learning
MLP	Multi-layer perception
OS	Overall survival
PFS	Progression-free survival
RFE	Recursive feature elimination
SIFT	Scale Invariant Feature Transform
TARGET	Therapeutically applicable research to generate effective treatment
TCGA	The cancer genome atlas databases
TDA	Topological data analysis
TERT	Telomerase reverse transcriptase
TMA	Tissue microarrays
OVR	One-versus-rest
UMAP	Uniform manifold approximation and projection
